# Innate and Adaptive Immune Genes Associated with MERS-CoV Infection in Dromedaries

**DOI:** 10.3390/cells10061291

**Published:** 2021-05-23

**Authors:** Sara Lado, Jean P. Elbers, Martin Plasil, Tom Loney, Pia Weidinger, Jeremy V. Camp, Jolanta Kolodziejek, Jan Futas, Dafalla A. Kannan, Pablo Orozco-terWengel, Petr Horin, Norbert Nowotny, Pamela A. Burger

**Affiliations:** 1 Research Institute of Wildlife Ecology, Department of Interdisciplinary Life Sciences, University of Veterinary Medicine Vienna, 1210 Vienna, Austria; sara.lado@vetmeduni.ac.at (S.L.); JeanPierre.Elbers@vetmeduni.ac.at (J.P.E.); 2 Department of Animal Genetics, University of Veterinary Sciences Brno, 61242 Brno, Czech Republic; m.plasil@gmail.com (M.P.); jfutas@vfu.cz (J.F.); horin@ics.muni.cz (P.H.); 3 RG Animal Immunogenomics, Ceitec Vetuni, 61242 Brno, Czech Republic; 4 College of Medicine, Mohammed Bin Rashid University of Medicine and Health Sciences, Dubai 505055, United Arab Emirates; tom.loney@mbru.ac.ae (T.L.); Norbert.Nowotny@vetmeduni.ac.at (N.N.); 5 Viral Zoonoses, Emerging and Vector-Borne Infections Group, Institute of Virology, University of Veterinary Medicine Vienna, 1210 Vienna, Austria; Pia.Weidinger@vetmeduni.ac.at (P.W.); Jeremy.Camp@meduniwien.ac.at (J.V.C.); Jolanta.Kolodziejek@vetmeduni.ac.at (J.K.); 6 Center for Virology, Medical University of Vienna, 1090 Vienna, Austria; 7 Al Ain City Municipality, Al Ain 15258, United Arab Emirates; Dafalla.Ahmed@aam.gov.ae; 8 The Sir Martin Evans Building, Cardiff School of Biosciences, Cardiff University, Museum Ave, Cardiff CF10 3AX, UK; Orozco-terWengelPA@cardiff.ac.uk

**Keywords:** coronavirus, immune response genes, Old World camels, in-solution hybridization capture, zoonosis

## Abstract

The recent SARS-CoV-2 pandemic has refocused attention to the *betacoronaviruses*, only eight years after the emergence of another zoonotic *betacoronavirus*, the Middle East respiratory syndrome coronavirus (MERS-CoV). While the wild source of SARS-CoV-2 may be disputed, for MERS-CoV, dromedaries are considered as source of zoonotic human infections. Testing 100 immune-response genes in 121 dromedaries from United Arab Emirates (UAE) for potential association with present MERS-CoV infection, we identified candidate genes with important functions in the adaptive, MHC-class I (*HLA-A-24*-like) and II (*HLA-DPB1*-like), and innate immune response (*PTPN4, MAGOHB*), and in cilia coating the respiratory tract (*DNAH7*). Some of these genes previously have been associated with viral replication in SARS-CoV-1/-2 in humans, others have an important role in the movement of bronchial cilia. These results suggest similar host genetic pathways associated with these *betacoronaviruses*, although further work is required to better understand the MERS-CoV disease dynamics in both dromedaries and humans.

## 1. Introduction

Emerging zoonotic diseases pose a serious threat not only to animal populations, but also to humans around the globe, as we experience with SARS-CoV-2 and the current COVID-19 pandemic [1]. A recent example of an emerging zoonotic pathogen in the family *Coronaviridae* is the Middle East respiratory syndrome coronavirus (MERS-CoV). It was first isolated in June 2012 from the sputum of a 60-year-old man from Saudi Arabia with acute pneumonia [2]. However, the April 2012 outbreak of acute respiratory illness in Jordan was retrospectively also diagnosed as MERS-CoV epidemic [3]. Similar to other emerging human coronaviruses, MERS-CoV is thought to have originated from bats; however, dromedaries (*Camelus dromedarius*) have been identified as reservoir hosts and the primary source of human infections [4,5,6,7]. This *betacoronavirus*, similar to the severe acute respiratory syndrome coronaviruses SARS-CoV-1 and SARS-CoV-2, affects the respiratory tract in humans yielding generalized symptoms typical of acute respiratory viral infections. While the infection takes a mild course in dromedaries, ranging from asymptomatic to minor naso-ocular discharge [5,8,9], humans often suffer from a severe course of disease with a fatality rate of up to 35% [2,10]. 

MERS-CoV in dromedaries was retrospectively traced back to at least 1992, as specific antibodies were detected in dromedary blood samples collected from different African regions in that year [11]. The asymptomatic or mild course of the disease in camels suggests that MERS-CoV has never had a major impact on dromedaries. Meanwhile, MERS-CoV infection in dromedaries has been confirmed in more than 25 countries on all continents except Australia [7]. Recently, also Bactrian camels (*Camelus bactrianus*) and hybrids between dromedary and Bactrian camels have been identified as potential reservoirs of MERS-CoV [12]. New World camelids (alpacas (*Vicugna pacos*) and llamas (*Lama glama*)) are also susceptible to the virus [13].

Camelids (family Camelidae) are recognized not only as multipurpose livestock adapted to extreme environments, producing milk, meat and wool under harsh conditions, but also for their potential in combating infectious diseases. Camelids are unique among mammals in their ability to generate homodimeric immunoglobulins (Ig) in addition to conventional antibodies, which usually consist of two heavy and two light chains. In all New and Old World camel species, the antigen-binding fragment of these specific IgGs is reduced to a single variable domain lacking the light chain, which significantly reduces the size of the antibodies [14,15,16]. The so-called “nanobodies” can be used for human clinical applications by transporting therapeutic agents into different body parts, also crossing the blood–brain barrier [17]. Recent evidence has revealed that camelids can produce nanobodies that effectively neutralize *betacoronaviruses* [18,19] and block SARS-CoV-2 infection [20], which makes them promising candidates for antiviral (i.e., COVID-19) therapy.

Although much information is available on MERS-CoV prevalence, epidemiology, genetic diversity [21], and etiopathology from experimental infections of dromedaries [22], little is known about the immune responses of camels to this zoonotic pathogen and its underlying genetic basis. The genetic knowledge gap might be partially due to the fact that high quality chromosome-assembled genomes have only become available very recently [16,23,24]. Indeed, genomic approaches would be a helpful tool for immune studies of non-model animals [25]. However, only a few genome-wide analyses have been performed in Old World camels, and these have mainly addressed genomic diversity and selection during domestication [16,26]. Moreover, there is a dearth of research investigating genotype–phenotype associations, and recent studies were targeted to blood parameters [27] or production traits [28]. Immunogenetic studies in camelids have either characterized important candidate genes for the adaptive and innate immune response, such as the major histocompatibility complex [29,30], natural killer cells [31] and T-cell receptors [14], or identified genomic organization and diversity of immune response (IR) genes in few individuals [16,24]. Consequently, increased knowledge about the immune system in Old World camelids and its genetic basis will further improve our understanding of their role in spill-over of MERS-CoV to humans [22].

We decided to use a target enrichment approach with in-solution hybridization capture of 100 annotated immune genes [24] to genotype a larger number of dromedaries tested for a recent infection of MERS-CoV. Targeted enrichment has successfully been applied in sequencing the IR genes of gopher tortoises [32], in assessing variants in immune genes associated with Hepatitis B virus infection [33] and in identifying somatic alterations in follicular dendritic cell sarcoma in known cancer-associated genes [34].

In this study, we aimed to characterize the diversity in IR genes and to detect variants potentially associated with MERS-CoV infection in dromedaries. We sequenced 100 IR genes using targeted enrichment via in-solution hybridization capture in 121 dromedaries collected in the field from three different sites in the United Arab Emirates (UAE). All dromedaries were assessed for MERS-CoV antibodies accounting for (past) infection by Enzyme-linked Immunosorbent Assay (ELISA) and Indirect Immunofluorescence Test (IIFT), as well as for the presence of the active virus (shedding) via molecular detection of viral nucleic acid using reverse transcription quantitative (RT-q) PCR. Most of the 121 dromedaries harbored MERS-CoV antibodies, which revealed that even young animals from the age of 2 months had already been exposed to the virus. Thus, we split all individuals positive for antibodies (seropositive) into two groups based on (i) virus presence (infection at the time of testing) and (ii) virus absence (no detectable infection at the time of testing). We also recorded the clinical status of the sampled dromedaries, however, due to the nonspecific signs of MERS-CoV, i.e., mild naso-ocular discharge, the clinical observations of the individuals were not convincing. As we are aware of the limitations related to a study conducted in the field (versus a controlled experiment), we accounted for (i) population structure, (ii) equal possibility of exposure to virus infection, and (iii) age and sex to reduce potential biases. Using phenotype–genotype association tests with univariate logistic regression, we identified candidate genes, which might be related to MERS-CoV infection in dromedaries. Although some of the gene variants associated to the presence of the disease have previously been related to SARS-CoV-1/-2 and other respiratory infectious diseases, further genomic and functional analyses will be necessary to broaden and corroborate our results. With our work, within all its limitations, we open doors for future novel research including large-scale screening for genes underlying defense mechanisms against an important zoonotic disease.

## 2. Materials and Methods

### 2.1. Ethics Statement

This study was approved by the Al Ain City Municipality and was part of ongoing public health surveillance in the UAE. The study was performed in accordance with the relevant laws and regulations that govern research in the UAE.

### 2.2. Sampling

The material for this study was collected during two field seasons (March/April 2019 and October 2019) from a total of 121 dromedaries, in three locations in the UAE: (1) the largest national livestock market (April 2019, *n* = 37; October 2019, *n* = 39); (2) a desert wildlife reserve (April 2019, *n* = 30); (3) a Bedouin family-owned farm (Al Mazrooei, Dubai, UAE) with camels primarily raised for racing and trading (March 2019, *n* = 15) (Table S1). Nasal swabs in RNA/DNA shield (ZymoResearch, Irvine, CA, USA) and serum samples of all dromedaries were collected and stored at −80 °C at the laboratory of the College of Medicine, Mohammed Bin Rashid University of Medicine and Health Sciences, Dubai, UAE before shipment to the University of Veterinary Medicine Vienna, Austria. As backup, we also collected tail hair samples, which were stored in labeled paper envelopes. All dromedaries (aged ≥ 6 months) in the UAE have a subcutaneous identity microchip that is linked to a national database containing information on the camel’s age, sex, and geographic origin within the UAE. All camels were scanned for these microchips and demographic data were extracted from the national database.

### 2.3. MERS-CoV Characterization

All nasal swab and serum samples were screened for MERS-CoV specific RNA. After thawing the samples were vortexed and centrifuged for 3 min at 6000 rpm. For automatic extraction with QIAcube (for 12 samples) or QIAcube HT (plate format device, both QIAGEN, Hilden, Germany) 140 or 200 µL, respectively, of each supernatant were taken. MERS CoV RT-qPCR in ORF1a gene region was performed using primers and probe as described previously [35]. However, this assay was adapted for qScript XLT One Step RT-qPCR ToughMix (QuantaBio, Beverly, MA, USA) on Applied Biosystems 7300 or 7500 Real-Time PCR systems (both Foster City, CA, USA). Samples with a Ct value equal or below 39.5 were considered positive.

To determine the presence of antibodies of immunoglobulin class IgG, IgA and IgM against MERS-CoV in vitro, serum samples were thawed, briefly centrifuged and subsequently screened using two camel specific serological assays: the Anti-MERS-CoV ELISA and the Anti-MERS-CoV IIFT (both EUROIMMUN, Lübeck, Germany) following the manufacturer’s instructions. For the ELISA, samples with an extinction ratio higher than 1.1 in relation to the calibrator were considered positive, samples with an extinction ratio lower than 0.8 were considered negative, and samples within 0.8–1.1 were regarded as borderline. For the IIFT, antibody titers were determined according to the fluorescence of the different sample dilutions (1:10–1:1000). Here, the manufacturer’s suggestions were adapted slightly to allow for a more conservative interpretation of the results (Table S2).

### 2.4. Camel DNA Extraction

DNA was extracted from a total of 82 nasal swabs, 11 hair and 28 blood samples with an improved salting-out method for high DNA yield [36] following a safety protocol (biosafety cabinet, FFP2 masks). DNA quantity was assessed by a spectrofluorometric assay using a fluorescence microplate reader (Twinkle; Berthold Technologies, Oak Ridge, TN, USA). Around 1 µg DNA from all 121 samples was sent to Daicel Arbor Biosciences (Ann Arbor, MI, USA) for library construction, hybridization capture and sequencing.

### 2.5. Probe Design and In-Solution Hybridization Capture Target Enrichment

We used a target enrichment approach based on in-solution hybridization with biotinylated RNA probes and selected 100 IR genes (Table S3) from the most up-to-date dromedary (CamDro3) annotations (https://doi.org/10.5061/dryad.qv9s4mwb3 (accessed on 1 February 2021); [23]) for myBaits^®^ design. The selected regions were provided to Daicel Arbor Biosciences (Ann Arbor, MI, USA) for bait design. A final total of 19,207 120-bp baits passed “Relaxed BLAST” analysis. For each 120-bp bait candidate, one BLAST hit against CamDro3 with the highest melting temperature was first discarded from the results (allowing for 1 hit in the genome), and only the top 500 hits (by bit score) were considered. Based on the distribution of remaining calculated melting temperatures, Daicel Arbor Biosciences filtered out nonspecific baits using the “Relaxed” (more nonspecific baits pass) criteria. Additional candidates were retained if they had at most 10 hits between 62.5 and 65 °C and 4 hits above 65 °C, and fewer than 2 passing baits on each flank.

Samples were sonicated and size selected following a protocol to produce an average insert length of approximately 300 bp. Up to 200 ng of sonicated and size selected DNA was taken into a library preparation method optimized for targeted capture. Unique dual-index combinations were added to each sample via 5–10 cycles of PCR amplification. The indexed libraries were quantified with both a spectrofluorimetric assay and a quantitative PCR assay. To prepare for capture, up to 80 ng of each library was pooled for capture (16- or 17-plex captures) and dried down to 7 µL by vacuum centrifugation. Captures were performed following the myBaits v4 protocol with an overnight hybridization. For each sample, half of the volume of beads in the elution buffer were amplified for 10 cycles. Final capture pools were quantified again with both a spectrofluorimetric and a quantitative PCR assay. Samples were sequenced on the Illumina NovaSeq 6000 platform on partial S4 flowcell lanes with 150-bp paired-end sequencing.

### 2.6. Variant Calling

We performed adapter and quality trimming using BBDuk v.38.75 (https://sourceforge.net/projects/bbmap/ (accessed on 5 February 2020)), using “ref = resources/adapters.fa” that comes with BBMap/BBTools v. 38.75. For this, we selected the following settings: ktrim = r, k = 23, mink = 11, hdist = 1, tpe, tbo, qtrim = rl, trimq = 15. We then mapped quality and adapter trimmed reads to the CamDro3 ([23]; https://doi.org/10.5061/dryad.qv9s4mwb3) assembly using BBMap v. 38.75 (https://sourceforge.net/projects/bbmap/) with the “usejni = t” setting. BAM files were cleaned, sorted, read groups added, and duplicates marked with Picard v. 2.21.7 (http://broadinstitute.github.io/picard). We called SNPs against CamDro3 [24] with CallVariants v. 38.39 (https://sourceforge.net/projects/bbmap/), keeping only SNPs with quality scores greater than or equal to 27 using the settings “ploidy =2 multisample minscore = 27.0 nopassdot = t duplicate = f minreadmapq = 30”. We then used BCFTools 1.9 (http://samtools.github.io/bcftools/ (accessed on 12 March 2019)) to filter each individual’s raw VCF file to exclude sites with missing genotypes, kept only SNPs that passed “CallVariants”‘s filters, and if a site was multiallelic, kept the genotype with the highest quality score. We also used BCFTools to merge VCF files for each individual into a single VCF file and finally employed BEDTools 2.29.0 [37] to keep only the SNPs that occurred in the target region where the 120-bp baits mapped using blastn v. 2.2.31+ [38].

### 2.7. Read-Based Imputation

We used STITCH v. 1.6.3 [39] to perform read-based imputation of SNPs. For this, first we selected scaffolds > 1 SNP and then extracted the positions of the SNPs. We performed adapter and quality trimming as before, and then we mapped the reads to CamDro3 with BBWrap v. 38.81 (https://sourceforge.net/projects/bbmap/) with “usejni = t” and “sam = 1.3” (output in the SAM 1.3 not 1.4 format for compatibility with STITCH 1.6.3). We finally ran STITCH 1.6.3 using the following number of ancestral haplotypes “k = {4, 6, 8, 10, 12, 14}” and the following number of generations ago “nGen = {100, 1000, 10,000, 100,000}” for each value of k and nGen, keeping only SNPs with INFO_SCORE > = 0.3. To determine the best combination of values of number of ancestral haplotypes and number of generations ago, we used JVarkit’s “vcfcomparegt” version deaac59 [40] to compare non-imputed (CallVariants 38.75) and imputed (STITCH 1.6.3) genotypes from Drom1829 (which had the most non-imputed genotypes). We chose k=14 and nGen=100000 with best performance showing the highest number of genotypes that were the same between Drom 1829 imputed and Drom 1829 non-imputed sample (see Table S4 for full results). We filtered STITCH SNPs with k = 14 and nGen = 100,000 with VCFTools 0.1.15 with “max-missing 0.90”, “min-alleles 2”, “max-alleles 2” and to retain only SNPs with allele frequency < 1 (polymorphic sites). Our imputed dataset contained 3958 SNPs for 121 dromedary samples.

### 2.8. Data Filtering

Quality control of the data with ≤10% missingness after read-based imputation was performed with PLINK 1.9 [41]. Relatedness was considered to detect samples with unexpectedly high value of identity by descent (IBS; i.e., >0.90) calculated in PLINK with the flags “--cluster” and “--matrix” to obtain the IBS matrix. We also filtered for a minor allele frequency (<1%) using the flag “--maf” and Hardy Weinberg equilibrium “--hwe” (*p*-value = 0.0000127, after FDR correction using “p.adjust” function on R 3.6.3 (R core team, 2019) based on the number of SNPs [42]).

### 2.9. Heterozygosity Associated in the Immune Response Genes

To assess the heterozygosity in the IR genes we used the GenomeTools v.1.5.8 [43] with the gff3 function and the ‘addintrons’ and ‘retainids’ options to predict intron regions from CamDro3 annotations [24]. We used BedTools 2.29.2 [37] to obtain only SNPs in exons, introns, or entire gene regions (it is possible for a SNP to be in both exon and intron regions of a gene, as transcript variants (isoforms) can differ by exon regions. We used Hierfstat 0.04-22 [44] with R 4.0.2 (R core team, 2020) to calculate observed (*H*_O_) and expected heterozygosity (*H*_E_) for each gene for exons, introns, and entire gene regions. We performed the same process to assess *H*_O_/*H*_E_ in the total dataset, in positive and negative individuals, as well as in the different sampling sites. We assessed normality of residuals with the shapiro.test function and homogeneity of variance using the base R 4.0.2 (R core team, 2020) with the R package Car 3.0-10 [45]. We used a Welch *t* test (implemented in the base R “t.test” function) for testing *H*_O_ and *H*_E_ significance in genes, coding (exons) and noncoding (introns) regions between cases and controls, without distinguishing gene groups. We used the base R lm and ANOVA functions to assess significance of main effects (*H*_O_ or *H*_E_ ~ Gene_Group) for gene, exon, and intron regions separately. If the Gene_Group was significant at the 0.05 level, we performed posthoc tests with Benjamini–Hochberg correction [42] with the R package multcomp version 1.4-15 [46].

### 2.10. Univariate Logistic Regression Analysis for Phenotype–Genotype Association

The association of SNPs (passing filtering criteria stated above) with the phenotype MERS-CoV-positive (case) and negative (control) was tested by univariate logistic regression analysis, accounting for sex, age and population structure. First, we included the first most informative PCs as covariates using PLINK 1.9 with the flags “--pca”. After, we used again PLINK 1.9 to perform the univariate logistic regression analysis by using “--logistic”, “--covar” and “--adjust”. Genomic inflation factor λ (lambda) was calculated in PLINK after applying logistic regressed *p*-values, and for values lower than 1, we calculated lambda on R (R core team). Graphical representations of Manhattan and Quantile-Quantile (QQ) plots were obtained with the R packages qqman v.0.1.4 [47] and ggbio v.1.36.0 [48]. We identified significant SNPs on a cut-off of *p* < 0.05 corrected for FDR [49]. Further SNPs located in genes with potential association with MERS-CoV infection were ranked by the lowest uncorrected significant *p*-values [50]. Gene names are based on functional annotations from Lado et al. ([25]; https://doi.org/10.5061/dryad.qv9s4mwb3), which we cross-referenced against GeneCards (https://www.genecards.org/). Finally, we used PLINK 1.9 to estimate allele frequencies and genotype counts, as well as to assess the significance differences, by using Fisher’s exact test with “--fisher” and “--model” “GENO” for allele frequencies and genotype counts between positives and negatives, respectively. We applied the “case-control for distinct traits” module in the Genetic Power Calculator ([51]; https://zzz.bwh.harvard.edu/gpc/ (accessed on 12 May 2021)) and estimated the minimum required sample size to achieve adequate statistical power (80%; alpha = 0.05; standard allelic test) for detecting evidence of an association in a candidate gene with significant minor allele frequency differences between cases and controls.

### 2.11. Linkage Disequilibrium (LD)-Based Gene-Set Test

We also performed a LD-based gene-set association analysis with PLINK v 1.9, using the SNPs in each of the 100 IR gene as a separate set. Set-based tests are particularly suited for large-scale candidate gene studies as opposed to whole genome association studies, as they can use permutation more efficiently. The empirical *p*-values were corrected for the multiple SNPs within a set (taking account of the LD between these SNPs). For this analysis we applied the default values of the standard r-squared (--set-r2) = 0.5, *p*-value (--set-*p*) = 0.05, max number SNPs (--set-max) = 5, and 10,000 permutations, representing a moderate setting of values.

## 3. Results

### 3.1. MERS-CoV Shedding and Antibody Prevalence in Dromedaries from the UAE

In this phenotype–genotype association study we investigated 121 dromedaries from three sites in the UAE: the largest national livestock market in the emirate of Abu Dhabi (*n* = 76), a desert wildlife reserve “Dubai Desert Conservation Reserve” ~60 km south-southeast of Dubai (*n* = 30), and a Bedouin owned camel farm (*n* = 15) ~70 km south of Dubai. Sex was equally represented within the samples (56 males, 57 females, 8 unknown sex) and the ages ranged from 2 months to almost 30 years (Table S1). We detected 107 and 117 seropositive dromedaries with MERS-CoV-specific Igs (IgG, IgA or IgM) by ELISA and IIFT, respectively, showing that most of these animals experienced a (past) MERS-CoV infection. Viral nucleic acids were detected by RT-qPCR in nasal swabs of 44 individuals (out of 76; 57.9%) from the livestock market exclusively (Table S1), including 18 females, 23 males and 3 unknown sex with an age between 2 months and 6 years. Almost half of the dromedaries (*n* = 37, 48.7%) were both RNA- and IgG-positive, which shows animals were infected and were likely shedding virus due to new (re)infection or persistent infection, possibly with continuous or intermittent shedding. Three MERS-CoV RNA-positive dromedaries were negative by both serological assays indicating recent virus infection of these animals. Three further RNA-positive animals were ELISA negative (or borderline positive) but IIFT positive. Nasal swabs from dromedaries from the two other sites (wildlife reserve and camel farm), as well as all serum samples tested negative by the MERS-CoV specific RT-qPCR (Table S1).

### 3.2. In-Solution Hybridization Capture and Variant Calls in Dromedary IR Genes

For the 1,305,546 base target region composed of exons and introns of 100 IR genes, we generated 823,887,778 reads (121, 136, 372, 601 bases) passing filter of which 82.99% reads were not PCR or optical duplicates. Of the unique reads, 97.53% were successfully aligned to the most up-to-date dromedary (CamDro3) reference genome [23] (mean ± standard deviation aligned reads per sample = 5,511,298 ± 1,534,987; minimum–maximum: 209,624–9,621,299). Mean coverage in the target region was generally high for each sample (186× ± 60×; minimum–maximum: 6×–320×), resulting in the identification of 5768 raw SNPs in the target region. After variant filtering, filtering for genotype missingness (<25%) and removing non-polymorphic loci, we identified 760 SNPs. Due to the low number of SNPs in the target region after filtering we performed read-based imputation, which has successfully been used by animal and plant breeders [52]. Imputation enabled extending the set of SNPs identified to 5730 SNPs, however, upon filtering these markers with INFO_SCORE ≥ 0.3 and removing non-polymorphic loci 3958 SNPs remained for further analyses.

We controlled for relatedness in the imputed dataset and no pair of individuals showed identity by descent higher than 0.88. Due to the high number of seropositive individuals, we continued the phenotype–genotype association analysis using univariate logistic regression including 101 dromedaries with antibodies confirmed by both ELISA and IIFT, after removing 14 borderline/negative samples for antibody prevalence, as well as three samples each with ambiguous virus infection results and missing age information. The seropositive dromedaries were split into two groups showing MERS-CoV presence (cases; *n* = 36) or absence (controls; *n* = 65). For the genotype data, we applied additional filtering steps to further reduce the possibility of capturing false positive variants and removed 13 SNPs out of Hardy–Weinberg equilibrium (HWE) exact test as well as 1003 SNPs with low minor allele frequencies of 1% or less (MAF < 1%). The final dataset consisted of 2942 SNPs genotyped in 101 dromedaries including 54 females, 46 males and 1 unknown, grouped into 36 cases and 65 controls.

### 3.3. Diversity in the Targeted Immune Response (IR) Genes

**Figure 1:**
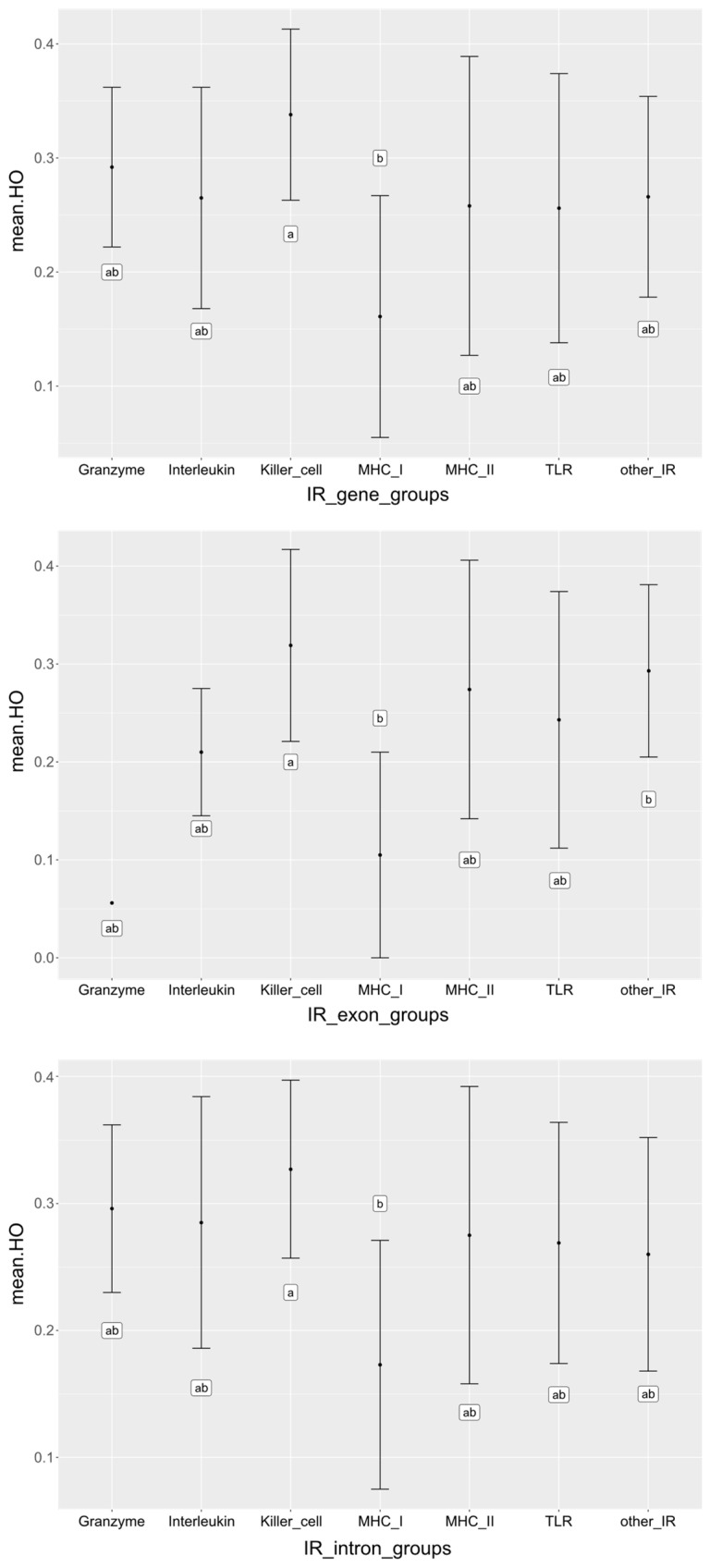
Observed heterozygosity (*H_O_*) values of immune response gene groups. Means and standard deviations are shown for predicted genes, exon and introns separately. Results are only presented for gene, intron and exon *H_O_* as only these showed significance for both ANOVA and posthoc correction with Benjamini–Hochberg (BH). Gene groups with different letters (‘a’ and ‘b’) indicate groups that had significantly different means whilst the same letters indicate nonsignificant different means.

To better understand the diversity of the 100 targeted IR genes, we organized them into functional groups, i.e., genes encoding MHC class I molecules, MHC class II molecules, toll-like receptors (TLR), granzymes, interleukins, genes expressed in natural killer (NK) cells (including natural killer cell complex (NKC) encoded killer cell lectin-like receptor (KLR) genes), and “other IR genes”. We estimated observed (*H*_O_) and expected (*H*_E_) heterozygosities in entire predicted genes, exons and introns separately. The average values calculated over all genes in the different IR gene groups ranged between 0.161–0.338 (*H*_O_) and 0.193–0.343 (*H*_E_), with the highest diversity (*H*_O_) observed in entire killer cell genes, and the lowest in MHC class I genes (Table 1). The specific values estimated in the IR genes are provided in Table S3. ANOVA tests after posthoc correction with Benjamini–Hochberg (BH) only showed significant (*p* < 0.05) differences in the *H*_O_ between MHC class I and killer cell genes, while all other gene group comparisons were not significant (Figure 1, Table S5). Furthermore, no gene, intron or exon *H*_O_ or *H*_E_ differed significantly (*p* < 0.05) between MERS-CoV positive and negative individuals (Table S6).

### 3.4. Phenotype–Genotype Association in MERS-CoV Antibody-Positive Dromedaries

**Figure 2:**
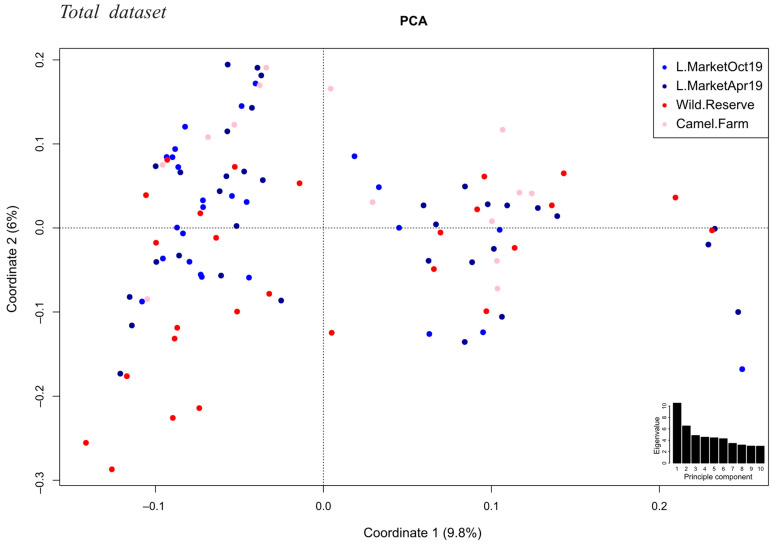
Principal component analysis of the population structure at three collection sites over two sampling periods. Variation explained by PC1 and PC2 are depicted in percentages. Individual animals are plotted on the first two principal components, colored by sampling site (livestock market (“L.Market”), over two sampling periods (April and October 2019, dark and light blue, respectively); Dubai Desert Conservation Reserve (“Wild.Reserve”), dark red; a Bedouin camel farm (“Camel.Farm”), pink). The inset shows a barplot of the eigenvalues for the first 10 principal components.

**Figure 3:**
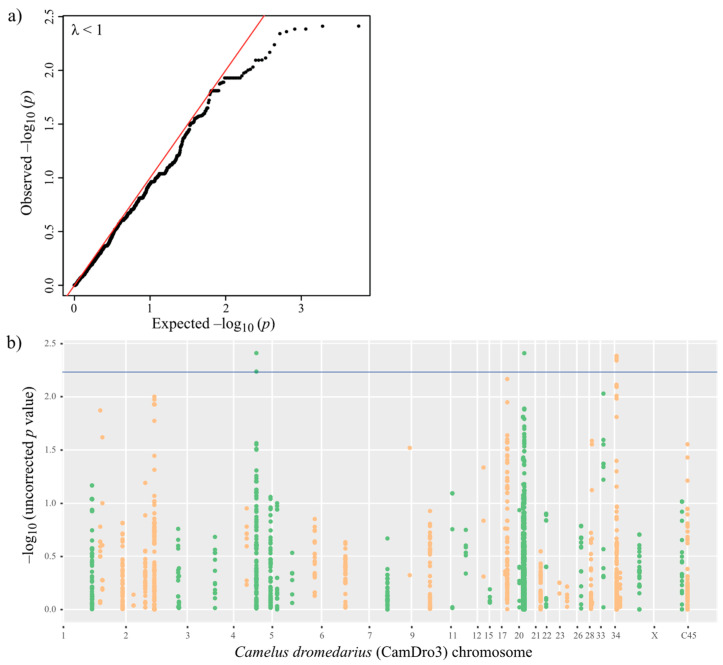
Univariate logistic regression results. (**a**) QQ plot, and (**b**) Manhattan plot with FDR threshold depicted in blue. −log_10_(*p*) values for SNPs alternate from green to orange to delineate chromosomes adjacent in the plots. C45 corresponds to Contig45, an unplaced scaffold in the CamDro3 reference.

As our samples originated from three different locations, we corrected for population structure to avoid population stratification bias and possible false positive associations. The genetic variation in the population explained by the first six most informative principal components (PCs 1–6) summed up to 33%, and we included these as covariates in addition to sex and age (Figure 2). We performed a univariate logistic regression with the complete dataset of 2942 SNPs imputed over 100 IR genes from 101 dromedaries seropositive for MERS-CoV antibodies, including 36 virus shedders (cases) and 65 virus nonshedders (controls). The genomic inflation estimation lambda (based on median chi square) was lower than 1 (λ = 0.82). The quantile-quantile (QQ) plot (Figure 3a) with PCA correction showed that in general observed values followed the expected values, with an end tail characteristic of SNPs in potential association with the tested phenotypes (MERS-CoV presence or absence) (Figure 3b for Manhattan plot).

The selection of an appropriate statistical significance threshold in phenotype–genotype association studies is critical to differentiate true positives from false positives and false negatives. Therefore, we decided to present significant markers that were selected based on a cut-off of *p* < 0.05 corrected for a false discovery rate (FDR; [49]). In addition, we present the most significant SNPs ranked by the lowest uncorrected *p*-values. We detected 16 candidate SNPs (uncorrected *p* < 0.01), of which the top seven were significant using the FDR corrected *p* < 0.0058, as displayed in the Manhattan plot (Figure 3b) and Table 2. The seven top candidate SNPs were located within three genes on chromosomes (chr) 5, 20 and 34, respectively: Protein Tyrosine Phosphatase Non-Receptor Type 4 (*PTPN4*), which contained two intronic SNPs; an MHC class I human leukocyte-associated antigen *(HLA) A-24*-like sequence with one SNP potentially in an intron close to exon 4. However, due to an equivocal annotation of this locus in the CamDro3 reference genome, it is not clear whether it is a complete, potentially functional MHC class I sequence, and *Mago Homolog B* (*MAGOHB*), which harbored four intronic variants. The other SNPs were found in introns of the genes Dynein Axonemal Heavy Chain 7 (*DNAH7*; chr 17), Interleukin 10 Receptor Subunit Alpha (*IL10RA*; chr 33) and Coiled-Coil and C2 Domain Containing 2A (*CC2D2A*; chr 2). Among the potentially associated variants with slightly higher *p*-values (≤0.01548), we identified nine SNPs (seven in introns, one in exon 2 and another one in exon 4) located in the MHC class II gene *HLA-DPB1*-like (Table 2), which we thus consider as a strong candidate as well.

Calculating allele frequencies for the top seven candidate SNPs, we detected significantly (*p* = 0.0028) higher frequencies of the minor allele (chr20:23100696) in the *HLA-A-24*-like sequence in MERS-CoV positive dromedaries (Table 2). We estimated the required sample size to detect evidence for an association with a power of 80% in this candidate locus at a minimum of 15 samples. In addition, the homozygote genotype counts for the minor allele in *MAGOHB* were significantly higher (*p* < 0.05) in MERS-CoV negative camels than in positive ones (Table 2). To further reduce the potential selection of false positive markers our next step was to test the robustness of these results by accounting for spatial and temporal sampling.

### 3.5. Accounting for Spatial and Temporal Sampling

We attempted to account for the limitation that dromedaries sampled from the three different locations might not have had equal exposure possibilities to MERS-CoV. Although MERS-CoV antibodies were present in all camels included in the association analysis, indicating they had contact with the virus, we only detected active virus infection (shedding) in camels sampled at the livestock market. In this largest national livestock market, dromedaries from all over the UAE are sold, and this cohort was less structured in terms of their exposure to infection. The other two sampling locations included a wildlife reserve where the camels do not have regular contact with other dromedaries and a Bedouin owned camel farm, where the animals are relatively isolated. As such, we repeated the univariate logistic regression analysis only including 60 samples from the livestock market (35 females, 24 males, 1 unknown), split into 36 cases (MERS-CoV positive) and 24 controls (MERS-CoV negative), and genotyped for 2917 SNPs (after filtering for HWE and MAF as before). As the dromedaries had been transported to the livestock market from all over the UAE, we corrected for potential population structure and included the first five most informative PCs (33% of the variation; Figure S1a) as a covariate in the univariate logistic regression analysis, along with sex and age. The genomic inflation estimation lambda (λ) was 0.78, and the QQ plot with PCA correction showed that observed values followed expected ones, with an end tail as observed in GWAS studies (Figure S2b). The most significant SNPs (Figure S2b) were located in the same four genes as detected in the initial test including complete dataset (101 individuals; 2942 SNPs).

Similarly, we accounted for the fact that we had two field seasons (spring and autumn 2019) and analyzed only those 75 samples collected in spring 2019 (36 females, 38 males, 1 unknown), divided into 22 cases and 53 controls. The univariate logistic regression with 3016 variants and PCs 1–6 (34.4%, Figure S1b) included as covariates resulted in a genomic inflation estimation lambda again lower than 1 (λ = 0.77) and a similar QQ plot (Figure S2c). Within the top most significant SNPs we identified three (*PTPN4*, *HLA-A-24*-like, *DNAH7*) out of the four previously detected genes (Figure S2c).

Finally, to better confirm the results from the imputed data set, we repeated the univariate logistic regression analysis with the initially called 760 SNPs after filtering for 25% of genotyping missingness (without imputation). Due to higher genotype missingness, we applied less stringent filtering for the relatedness threshold (0.95), but similar HWE (0.0000658) and MAF (<1%) thresholds as before, as well as taking into account population structure (first four PCs explaining 36% of the total variation). After filtering, 696 variants and 98 samples (35 cases and 63 controls) were included. With a lambda lower than 1 (λ = 0.66), *PTPN4* and *HLA-A-24*-like harbored the top SNPs (uncorrected *p* < 0.02) (Figure S2d).

### 3.6. Linkage Disequilibrium-Based Gene-Set Test

To further test the robustness of our results, we applied a complementary approach by means of a gene-set association test [41] using the complete dataset (101 individuals; 2942 SNPs). Overall, the gene-set results were similar to the univariate logistic regression SNP tests. From the 100 targeted IR genes, 20 had significant SNPs (uncorrected *p* < 0.05), including the genes *PTPN4*, *DNAH7*, *HLA-A-24*-like, *HLA-DPB1*-like, and *MAGOHB* (Table S7). *HLA-A-24*-like and *MAGOHB* were nominally significant (*p* = 0.031 and *p* = 0.008) and harbored 112 and 12 variants, of which 14 and 5 SNPs were significant, respectively. However, only two SNPs in *HLA-A-24*-like (chr20:23100696 | 23100503) and one in *MAGOHB* (chr34:15362634) passed the independent significance r-squared based threshold of 0.5. While these genes showed a stronger signal for potential genotype–phenotype association, none of the other genes were nominally significant (Table S7).

## 4. Discussion

It has long been established that a combination of population growth, biodiversity loss and land-use change drives the emergence and spread of zoonotic diseases [53]. The emergence of MERS-CoV over the past decade is no exception, being the likely outcome from such combined factors. As the consumption of camel milk and meat is increasing and camel products gain access to wider markets, the impact of camel-associated zoonotic diseases on public health and economics will also grow with advancing urbanization in African and Arabian countries. In this study we attend to this important zoonosis and target the immune response to MERS-CoV infection in a representative dromedary sample from the UAE.

### 4.1. High MERS-CoV Antibody Prevalence in Dromedaries from the UAE

In the course of this study, the assessment for the presence of MERS-CoV antibodies revealed a high prevalence (88%) of seropositive individuals (*n* = 107) within the 121 investigated dromedaries from the UAE. Additionally, another 94 dromedaries were screened as part of a public health surveillance and all showed seropositivity (N. Nowotny, personal communication). While sex was equally distributed over seropositive individuals in our study, age was as young as 2 months with an average of 6 years (Table S1). Previous studies have shown that seropositivity is higher among adult dromedaries (2 years and older), indicating that the likelihood of exposure and subsequent infection increases with age [54]. Camels generally shed MERS-CoV for about 7 days and viral RNA is detectable with RT-qPCR up to 35 days post-infection. Virus-specific antibodies can be identified from 3 weeks after infection onwards, with anti-MERS-CoV IgMs present for at least 4 weeks (indicating a more recent infection), while anti-MERS-CoV IgGs can stay for many years [55]. High seroprevalence coupled with known instances of camel–human transmission provides a proxy for prospective epidemiological risks [56], as a human case study in Saudi Arabia with higher-than-expected prevalence of MERS-CoV seropositivity in dromedaries demonstrated [57]. Naturally infected dromedary camels shed virus from the upper respiratory tract, evidenced by the presence of RNA in the RT-qPCR from the nasal swabs we collected. No current MERS-CoV positives were detected among the Bedouin family-owned dromedaries or the wildlife reserve (Table S1), compared to the livestock market where animals from different regions in the UAE are mixed in medium-sized (~50–100 sqm) pens. 

### 4.2. Different Diversity in IR Gene Groups

We observed that MHC class I mean diversity (*H*_O_) was significantly lower compared to killer cell genes over all dromedaries. Low levels of genetic diversity in the MHC region have been also observed in wild and domestic two-humped camels [29]. Interestingly, a lower overall genomic heterozygosity was described in dromedaries compared to wild and domestic Bactrian camels [26], which could hint to a generally lower genetic diversity in dromedaries. However, recent genome-wide analyses of IR genes found that the mean nucleotide diversity in MHC class I and II genes in dromedaries and domestic Bactrian camels seemed to be higher compared to other adaptive or innate IR genes, as well as the rest-of-genome genes, at least for the MHC genes studied [24].

### 4.3. Candidate IR Genes Associated with MERS-CoV Infection in Dromedaries

Since 2002, three betacoronaviruses, i.e., SARS-CoV-1, MERS-CoV and the most recent SARS-CoV-2 emerged as human pathogens through possible zoonotic spill-over from animals, all associated with severe human respiratory infections. Both candidate gene and genome-wide sequencing approaches have offered relevant insights into the genetic basis of these zoonotic diseases [58]. Unlike for SARS-CoV-1 and -2, few studies have been conducted on host genetic variation underlying susceptibility to MERS-CoV, its pathogenesis, transmission, and mortality in humans [59,60]. In this field study, we identified candidate genes on four different chromosomes potentially associated to MERS-CoV infection in dromedaries from the UAE, *PTPN4*, *DNAH7*, MHC class-I (*HLA-A-24*-like sequence), MHC class II (*HLA-DPB1*-like) and *MAGOHB.* The SNPs significantly associated with the presence of MERS-CoV in seropositive camels were mainly distributed in intronic regions (Table 2) except for the MHC class II gene *HLA-DPB1*-like, where we found one SNP in exon 2 and another in exon 4 (Table 2). Exon 2 encodes the antigen-binding groove of the class II molecule and, therefore, its polymorphism is of functional importance. Exon 4 codes for the transmembrane domain that controls membrane domain partitioning and class II structure, both of which influence antigen presentation and T-cell activation [61]. In recent years, an important role of intronic polymorphisms has been established, either filling regulatory functions upstream of exons or being in linkage with other (exonic) variants (e.g., [62]). The *HLA-A-24*-like sequence harboring the significant chr20:23100696 and further variants (Table 2 and Figure S2) is incompletely annotated as fully functional classical MHC class I gene, with no exon 2 sequence, in the dromedary reference genome (CamDro3) as well as in the next closely related and chromosome-assembled genome of the wild camel (*Camelus ferus*; [16]). Therefore, we cannot exclude the possibility that we sequenced a pseudogene or a misassembled chimeric sequence for this locus. Although the (functional) impact of the identified candidate genes on MERS-CoV infection in dromedaries has yet to be determined, previous research associated those genes with viral replication in SARS-CoV-1/-2, MERS-CoV and other viruses causing respiratory infectious diseases in humans, and with pathways involved in the movement of bronchial cilia. 

*PTPN4*. *PTPN4* belongs to the protein tyrosine phosphatase (PTP) family and has an important role in the innate immune system. For instance, it inhibits the Toll-like receptor (TLR) 4 signaling pathway that triggers many immune proteins including proinflammatory cytokines and type I interferons [63]. Interestingly, while PTPN4 acts as an inhibitor, the TLR4 signaling pathway is activated by the SARS-CoV-2 spike protein [64], and mice lacking TLR4 had more severe SARS-CoV infections than wild-type mice [65]. Meanwhile, the spike protein of MERS-CoV triggers the expression of negative regulators of the TLR signaling pathways [65]. Understanding the TLR signaling pathways in the context of MERS-CoV infection also in dromedaries would be an important contribution to mitigate the viral infection. *PTPN4* is related to predicted target functions of human micro(mi)RNAs that bind to the single-stranded (ss)-RNA such as SARS-CoV-2, and possibly to its spike protein gene. These predicted miRNA targets might destabilize the ss-RNA translation of SARS-CoV-2 in respiratory epithelial cells, which could explain successful antiviral defense [66]. As both polymorphisms identified in the UAE dromedaries are located in intronic regions (introns 25 and 26) of *PTPN4*, they might have regulatory functions that can influence the expression of the gene [62].

*DNAH7. DNAH7* encodes a force-generating protein that is an essential component of the inner dynein arm of axonemes in cilia coating the respiratory tract, which drive mucus along airway surfaces providing a critical defense mechanism of the pulmonary system [67]. It represents one of the most downregulated genes following SARS-CoV-2 infection of human bronchial epithelial cells in vitro [68]. *DNAH7* expression levels were also significantly downregulated in human bronchial epithelial cells infected with MERS-CoV and influenza A (H1N1), which induce apoptosis in these cells [69,70]. To recognize if similar mechanisms act in dromedaries with MERS-CoV infection, specific gene expression studies including camel bronchial epithelial cells would be necessary.

*HLA-A-24*-like (MHC class I A-24). The MHC (classes I, II, and III) has a dense clustering of immune relevant genes that can show extreme polymorphisms due to their main task of encoding cell surface proteins involved in antigen presentation [71]. Thus, HLA polymorphisms have been linked to susceptibility and pathogenesis of numerous infectious diseases including those caused by RNA viruses, especially SARS, influenza, AIDS, rabies, and West Nile fever [59]. For example, a protective effect of HLA-A*02:01 against SARS-CoV-1 has been suggested in Asian patients [72,73], while HLA-A*24:02 has been associated with COVID-19 susceptibility [74]. Large meta-analyses of allele frequency distributions in human traits showed that SNPs connected to disease susceptibility are generally skewed towards a higher minor allele frequency (>20%) [75]. In our study, a significantly higher (25%; *p* = 0.0028) frequency was also observed for the minor allele of the *HLA-A-24*-like chr20:23100696 in MERS-CoV positive dromedaries (Table 2). However, as this result concerns only a single SNP within a sequence of unclear status, several different interpretations are possible, including a false positive statistical artifact, an isolated finding due to an inaccurate annotation and assembly of the sequence and/or an effect of linkage with a causative MHC SNP variant. Therefore, our observation that UAE dromedaries with higher MAF within the *HLA-A-24*-like sequence might be more susceptible to MERS-CoV infection needs to be corroborated by additional (long-read) sequencing and haplotype analysis, including samples from other Arabian and African populations.

*HLA-DPB1*-like (MHC class II DPB1). Associations observed for two MHC class II SNPs located in exons 2 and 4 support the idea of an MHC effect on the phenotypes analyzed. Although antigen presentation of SARS-CoV-1 mainly depends on MHC class I molecules [76], class II genes can also contribute to *Betacoronaviridae* antigen presentation as suggested by the association of HLA-DRB1*11:01 and HLA-DQB1*02:02 alleles with susceptibility to MERS-CoV [60]. In COVID-19 patients from Italy, the allele frequency distributions for HLA-DRB1*15:01 and DQB1*06:02 showed significant correlations of the minor allele with higher susceptibility to the disease, while DRB1*07:01 on the contrary was negatively associated [77]. Interestingly, dromedaries with no current MERS-CoV infection were more often homozygote for the minor allele of *HLA-DPB1*-like, which is a paralog of *HLA-DRB1* in humans. The associations observed may, however, result from the effect of linkage with other MHC sequences.

*MAGOHB. MAGOHB* belongs to the mago nashi gene family and is required for pre-mRNA splicing. In macrophages—one of the effector cells of the innate immune system—the expression of *MAGOHB* increased rapidly after lipopolysaccharide (LPS) stimulation [78]. LPS is a natural adjuvant, which is synthesized by Gram-negative bacteria, and stimulates cells through TLR4 signaling pathway, causing the release of inflammatory cytokines and the upregulation of costimulatory molecules on antigen presenting cells [79]. Interestingly, *MAGOHB* is targeted by has-miR-20a-5p, one of six miRNAs that previously have been reported to be antiviral in respiratory diseases, and were found to be downregulated in lung tissues during viral infection [80,81]. From a network analysis, has-miR-20a-5p was identified among 38 miRNAs targeting host genes that interact with SARS-CoV-2 proteins [82]. The homozygote alternative (minor) genotype of *MAGOHB* was significantly (*p* < 0.005) more frequent in our control group, which might hint to a higher resistance to MERS-CoV infection in these dromedaries. It is also possible that *MAGOHB* represents positional markers in linkage with some genes of the natural killer complex (NKC). The dromedary NKC harbors, besides tested KLR genes, a number of other C-type lectin-like (CLEC) receptor genes downstream of *MAGOHB* [31] that were not included in the hybridization capture of IR genes. CLECs are expressed by myeloid cells and serve to monitor their environment and sense danger. In principle, they recognize a vast repertoire of (non-)glycan ligands from pathogens or modulate activity of cells. Dendritic cell CLECs, for example, by recognition and internalization of ligands start the process of antigen presentation to T cells and generation of an immune response [83].

In summary, we identified important candidate genes related to the innate and adaptive immune system in dromedaries from the UAE. The functional importance of these genes in response to MERS-CoV infection in dromedaries, similar to humans, needs to be investigated in more controlled in vitro and in vivo experiments.

### 4.4. Challenges and Impact of IR Gene Associations with Betacoronavirus Infections

Biological interpretations of statistical significance in association (field) studies have several limitations. While we did our best to select an equal distribution of age and sex and to account for potential population structure in the dromedaries sampled from three locations in the UAE, we cannot exclude that some of the associations presented here are false positives. Due to the nature of a field study, we cannot guarantee that all individuals had equally been exposed to MERS-CoV, although we included only dromedaries with antibodies indicating that they have had contact with the virus at some time in the past. We attempted to control for this fact by repeating the univariate logistic genotype–phenotype regression analysis only with dromedaries from the largest livestock market located in the emirate of Abu Dhabi, where animals from all over the UAE are traded. Our different tests accounting for spatial and temporal sampling showed in general good coherence of the results in the top selected candidate genes. The genetic power calculation for the required sample size to detect “true” association with a power of 80% in a candidate gene with significant allele frequency differences between case and controls resulted in a minimum number of 15 samples, which was reached throughout all subsampling strategies (e.g., 22 cases in the spring season samples, Figure S1b). While the analyzed virus assessment reflects presence/absence of the virus in seropositive camels, the nonrandom distribution of SNPs observed between actively infected/noninfected individuals indicates that they are genetically different, which consequently demands further investigations especially in terms of their immune mechanisms. Future case–case control studies need to include more dromedary populations from different African and Arabian countries and, though challenging, potential birth cohort studies or experimental MERS-CoV inoculation of dromedaries and other livestock herded together. This would help define predisposed groups and support screening efforts for potential virus reservoirs. The next important step will be to investigate expression and functional pathways of the identified candidate IR genes to select for higher resistance to MERS-CoV. Finally, the innate and adaptive IR genes identified in dromedaries show high resemblances with human immune response to the zoonotic SARS-CoV-1, SARS-CoV-2 and MERS-CoV. Thus, understanding the underlying mechanisms to disease susceptibility/resistance in dromedaries and other animals will result in more effective strategies to combat *betacoronaviral* disease in human populations as well. 

## Figures and Tables

**Table 1:** Average observed (*H*_O_) and expected (*H*_E_) heterozygosity in the captured immune response gene groups estimated over all genes, exons and introns.

IR Gene Group	Genes ^a^			Exons ^a^			Introns ^a^		
	SNPs	*H*_O_	*H*_E_	SNPs	*H*_O_	*H*_E_	SNPs	*H*_O_	*H*_E_
Granzyme	54	0.292	0.311	5	0.056	0.066	50	0.296	0.315
Interleukin	121	0.265	0.284	21	0.210	0.218	107	0.285	0.305
Killer cells	282	0.338	0.342	38	0.319	0.313	253	0.327	0.336
MHC class I	435	0.161	0.193	96	0.155	0.185	353	0.173	0.205
MHC class II	596	0.258	0.277	140	0.274	0.290	456	0.275	0.294
TLR	124	0.256	0.298	55	0.243	0.272	74	0.269	0.311
Other IR genes	1253	0.266	0.280	235	0.293	0.307	1074	0.260	0.273

^a^ Genes, exons, or introns with fewer than two SNPs could not be included in calculations of averages.

**Table 2:** Significant SNPs located in candidate IR genes, allele frequencies of the minor alleles and genotype counts (homozygote minor/heterozygote/homozygote major allele) in MERS-CoV positive (cases) and negative (controls) camels.

Chr	Position (Minor/Major Allele)	Gene	Association Test *p*-Value	Allele Freq. Minor Allele				Genotype Counts		
				Cases	Controls	Exact *p*-Value	Odds Ratio	Cases	Controls	Exact *p*-Value
5	T8508361C intron 25	*PTPN4*	0.003873 **	0.08824	0.2	0.06096	0.3871	0/6/28	2/20/38	0.1241
20	A23100696G intron 4	*HLA-A-24*-like	0.003881 **	0.25	0.08475	0.00278 *	3.6	1/16/19	0/10/49	0.002554 *
34	G15362634A intron 1	*MAGOHB*	0.004123 **	0.3	0.4308	0.09343	0.5663	1/19/15	16/24/25	0.01201 *
34	A15363451G intron 2	*MAGOHB*	0.004129 **	0.2941	0.4308	0.06634	0.5506	1/18/15	16/24/25	0.01392 *
34	15367780	INTERGENIC	0.004364 **	-	-	-	-	-	-	-
34	G15361800A intron 1	*MAGOHB*	0.004553 **	0.3	0.4206	0.1239	0.5903	1/19/15	15/23/25	0.01467 *
5	C8506434T intron 26	*PTPN4*	0.005774 **	0.1	0.2097	0.07213	0.4188	0/7/28	2/22/38	0.1349
17	T23840747C intron 6	*DNAH7*	0.006791	0.1806	0.1154	0.2087	1.689	1/11/24	1/13/51	0.3252
34	C15363470T intron 2	*MAGOHB*	0.008005	0.3286	0.4385	0.173	0.6267	2/19/14	16/25/24	0.04715*
20	A20676706C intron 4	*HLA-DPB1*-like	0.01548	0.04167	0.09231	0.2651	0.4275	0/3/33	2/8/55	0.6674
20	T20677126C exon 4	*HLA-DPB1*-like	0.01548	0.04167	0.09231	0.2651	0.4275	0/3/33	2/8/55	0.6674
20	G20678240T intron 2	*HLA-DPB1*-like	0.01548	0.04167	0.09231	0.2651	0.4275	0/3/33	2/8/55	0.6674
20	T20679052C exon 2	*HLA-DPB1*-like	0.01548	0.04167	0.09231	0.2651	0.4275	0/3/33	2/8/55	0.6674
20	A20679884C intron 1	*HLA-DPB1*-like	0.01548	0.04167	0.09231	0.2651	0.4275	0/3/33	2/8/55	0.6674
20	G20680467A intron 1	*HLA-DPB1*-like	0.01548	0.04167	0.09231	0.2651	0.4275	0/3/33	2/8/55	0.6674
20	T20680474C intron 1	*HLA-DPB1*-like	0.01548	0.04167	0.09231	0.2651	0.4275	0/3/33	2/8/55	0.6674
20	T20680741C intron 1	*HLA-DPB1*-like	0.01548	0.04167	0.08594	0.3868	0.4625	0/3/33	2/7/55	0.6561
20	G20681619C intron 1	*HLA-DPB1*-like	0.01548	0.04167	0.09231	0.2651	0.4275	0/3/33	2/8/55	0.6674

* significant *p* < 0.05; ** significant after FDR correction.

## Data Availability

Raw FASTQ files were deposited on the European Nucleotide Archive (ENA) (ERS5621787 (SAMEA7874536)–ERS5621907 (SAMEA7874656)). VCF file, target region and bait sequences were deposited on dryad together with the scripts file (https://datadryad.org/stash/share/OfmXVPdiw1tJ0LxeMZ_b7gky8ty3F0BOqfnGwz1qf1I (accessed on 27 January 2021)). Additional material requests can be addressed to Pamela.Burger@vetmeduni.ac.at.

## Supplementary Materials

The following are available online at https://www.mdpi.com/article/10.3390/cells10061291/s1, Figure S1: Principal component analysis of the population structure at three collection sites over two sampling periods. Variation explained by PC1 and PC2 is depicted in percentages. Individual animals are plotted on the first two principal components, colored by sampling site (livestock market (“L.Market”) over two sampling periods (April and October 2019, dark and light blue, respectively); Dubai Desert Conservation Reserve (“Wild.Reserve”), dark red; a Bedouin camel farm (“Camel.Farm”), pink). The inset shows a barplot of the eigenvalues for the first 10 principal components. (a) Only livestock market samples; (b) only spring field season: (c) non-imputed dataset, Figure S2: Manhattan and QQ plot. Highlighted in bold are the four genes that are common in all analyses (*HLA-A*-like, *PTPN4*, *MAGOHB* and *DNAH7*). FDR corrected thresholds are represented in blue. (a) Total dataset; (b) only livestock market samples; (c) only spring field samples; (d) non-imputed dataset. C45 corresponds to Contig45, an unplaced scaffold, Table S1: Sample information and assessment of virus presence (swabs) and antibody prevalence (sera), Table S2: Scheme showing how IIFT antibody titers were determined according to the fluorescence of the different sample dilutions, Table S3. Observed (*H*_O_) and expected (*H*_E_) heterozygosity values depicted in immune response gene groups. Identified candidate genes *MAGOHB*, *HLA-A-24-like*, *HLA-DPB1-like*, *DNAH7* and *PTPN4* are highlighted in bold, Table S4. Read-based imputation performance. nAncestralHaplotypes (k) = number of ancestral haplotypes; nGen = number of generations ago, controls recombination rate; nDiff_from_non-imputed = number of genotypes that were not the same between imputed and non-imputed samples; nMatch_from_non-imputed = number of genotypes that were the same between imputed and non-imputed sample; nMissing_from_non-imputed = these are SNPs and hence genotypes that are missing because that SNP failed QC for imputation; nAdditional_SNPs_with_called_genotypes_from_non-imputed = these are genotypes newly added by imputation), Table S5: Statistical analysis of observed heterozygosity (*H*_O_) for immune response gene groups in genes, exons and introns. Means and standard deviations are shown for genes, exon and introns separately. Results are only presented for gene, intron and exon *H*_O_ as only these showed significance for both ANOVA and posthoc correction with Benjamini–Hochberg (BH). Gene groups with different letters (‘a’ and ‘b’) indicate groups that had significantly different means whilst the same letters indicate nonsignificant different means, Table S6: Observed (*H*_O_) and expected (*H*_E_) heterozygosity in genes, exons and introns in MERS-CoV positive (*n* = 36) and negative (*n* = 65) individuals. *p*-values of mean differences were calculated with Welch *t* test, Table S7: Linkage disequilibrium-based haplotype (gene-set) test showing 20 genes with significant SNPs at *p* < 0.05. Identified candidate genes *MAGOHB*, *HLA-A-24-like*, *HLA-DPB1-like*, *DNAH7* and *PTPN4* are highlighted in bold. *HLA-A-24-like* and *MAGOHB* were nominally significant (*p* < 0.05) indicated with an asterisk. NSNP—number of SNPs in set; NSIG—total number of SNPs below *p*-value threshold; ISIG—number of significant SNPs also passing LD-criterion; STAT—average test statistic based on ISIG SNPs; EMP1—empirical set-based *p*-value; SNPs—positions of SNPs in the set.
