# Supplementary material for: Innate and Adaptive Immune Genes Associated with MERS-CoV Infection in Dromedaries
Source: Cells. 2021 May 23;10(6):1291. doi: 10.3390/cells10061291 (PMC8224694; doi:10.3390/cells10061291)
Supplement: Supplementary file 1 [file cells-10-01291-s001.zip › cells-1174611-supplementary.pdf]

# Supplementary Materials for

## Innate and adaptive immune genes associated with MERS-CoV infection in dromedaries

Sara Lado, Jean P. Elbers, Martin Plasil, Tom Loney, Pia Weidinger, Jeremy V. Camp, Jolanta Kolodziejek, Jan Futas, Dafalla O. Kannan, Pablo Orozco-terWengel, Petr Horin, Norbert Nowotny, Pamela A. Burger*
*Corresponding author. pamela.burger@vetmeduni.ac.at

**This PDF file includes:**

Figures S1 and S2
Tables S1 to S7

a) *Only livestock market samples*

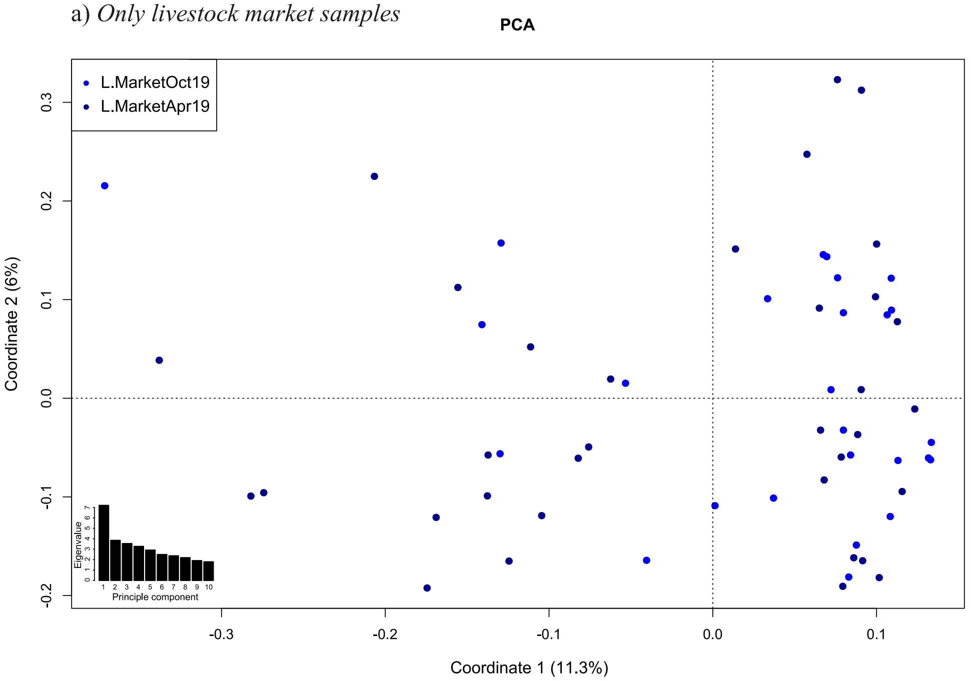


b) *Only spring field season*

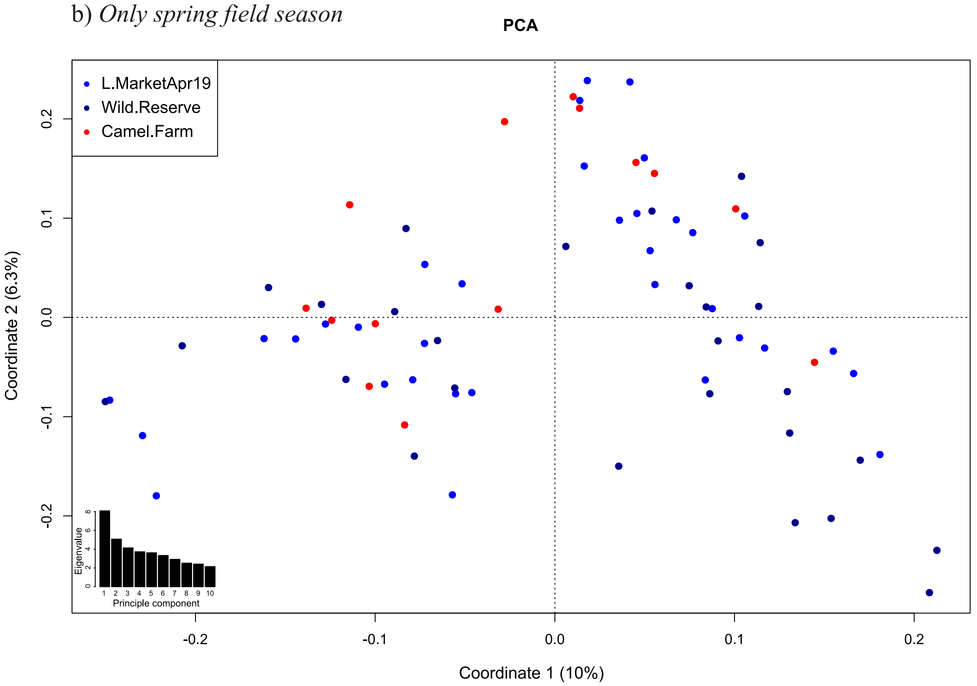

c) *Non-imputed dataset*

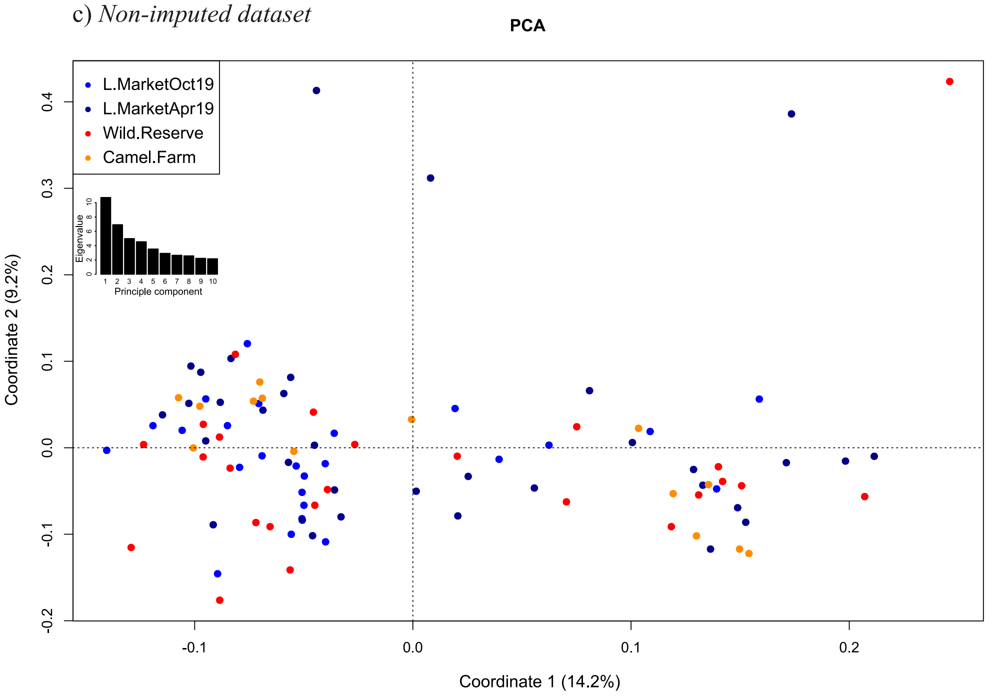


**Figure S1. Principal Component Analysis of the population structure at three collection sites over two sampling periods.** Variation explained by PC1 and PC2 are depicted in percentages. Individual animals are plotted on the first two principal components, colored by sampling site (livestock market [“L.Market”], over two sampling periods (April and October 2019, dark and light blue, respectively); Dubai Desert Conservation Reserve [“Wild.Reserve”], dark red; and a Bedouin camel farm [“Camel.Farm”], pink). The inset shows a barplot of the eigenvalues for the first 10 principal components. a) Only livestock market samples; b) Only spring field season: c) Non-imputed dataset.

a) PCA correction
(six first PC)

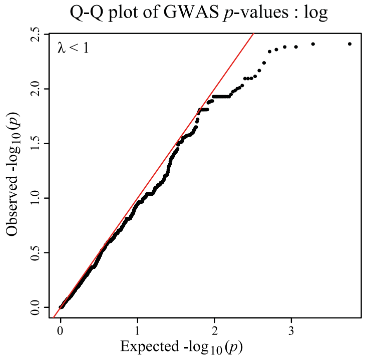


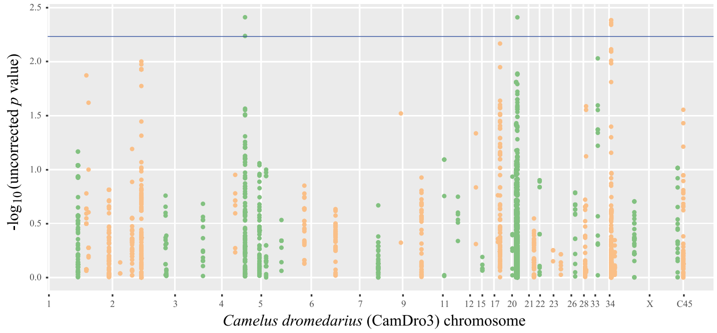


| Chr | Position | Gene | *p* - value |
|---|---|---|---|
| 5 | 8508361 | ***PTPN4*** | 0.0039 |
| 20 | 23100696 | ***HLA-A-24*** | 0.0039 |
| 34 | 15362634 | ***MAGOHB*** | 0.0041 |
| 34 | 15363451 | ***MAGOHB*** | 0.0041 |
| 34 | 15367780 | INTERGENIC | 0.0044 |
| 34 | 15361800 | ***MAGOHB*** | 0.0046 |
| 5 | 8506434 | ***PTPN4*** | 0.0058 |
| 17 | 23840747 | ***DNAH7*** | 0.0068 |
| 34 | 15371299 | INTERGENIC | 0.0077 |
| 34 | 15363470 | ***MAGOHB*** | 0.0080 |
| 34 | 15369030 | INTERGENIC | 0.0080 |
| 34 | 15371264 | INTERGENIC | 0.0080 |
| 33 | 12210072 | *IL10RA* | 0.0093 |
| 34 | 15370956 | INTERGENIC | 0.0098 |
| 2 | 113136710 | *CC2D2A* | 0.0099 |
| 34 | 15371123 | INTERGENIC | 0.0104 |

b) Only livestock market samples, with PCA correction
(five first PC)

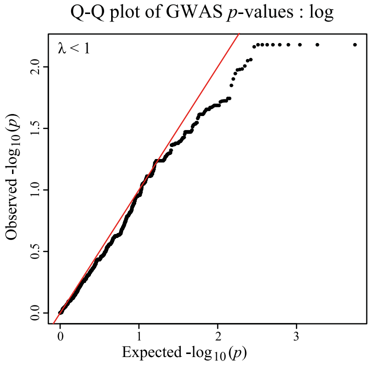


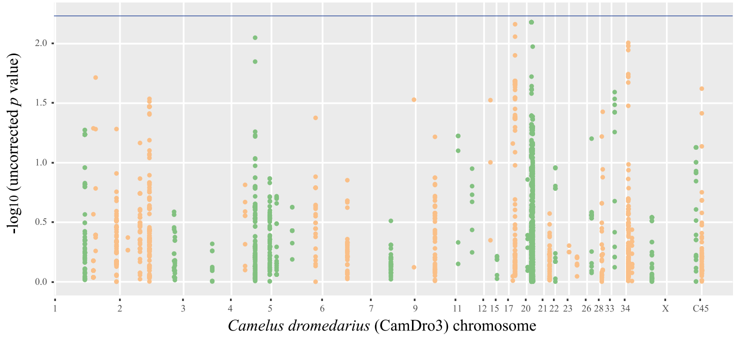


| Chr | Position | Gene | *p* - value |
|---|---|---|---|
| 20 | 20676706 | *HLA-DPB1* | 0.0066 |
| 20 | 20677126 | *HLA-DPB1* | 0.0066 |
| 20 | 20678240 | *HLA-DPB1* | 0.0066 |
| 20 | 20679052 | *HLA-DPB1* | 0.0066 |
| 20 | 20679884 | *HLA-DPB1* | 0.0066 |
| 20 | 20680467 | *HLA-DPB1* | 0.0066 |
| 20 | 20680474 | *HLA-DPB1* | 0.0066 |
| 20 | 20680741 | *HLA-DPB1* | 0.0066 |
| 20 | 20681619 | *HLA-DPB1* | 0.0066 |
| 17 | 23963829 | ***DNAH7*** | 0.0069 |
| 17 | 23948208 | ***DNAH7*** | 0.0087 |
| 5 | 8508361 | ***PTPN4*** | 0.0089 |
| 34 | 15363451 | ***MAGOHB*** | 0.0098 |
| 34 | 15362634 | ***MAGOHB*** | 0.0104 |
| 34 | 15367780 | INTERGENIC | 0.0105 |
| 20 | 23100696 | ***HLA-A-24*** | 0.0106 |

c) Only samples from spring field season, with PCA correction (six first PC)

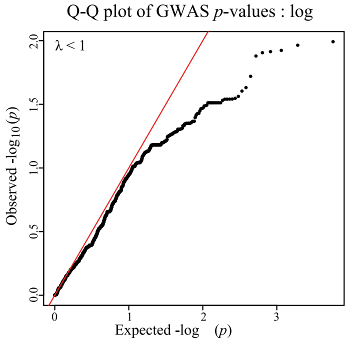


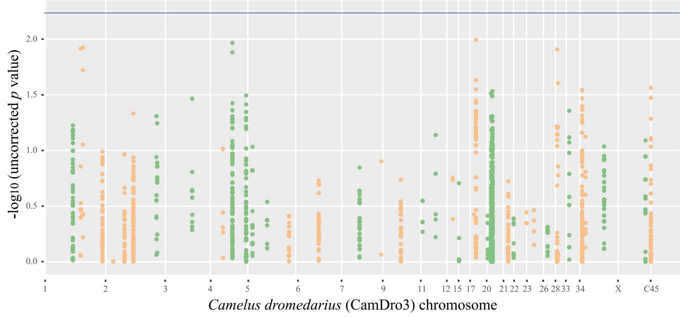


| Chr | Position | Gene | *p* - value |
|---|---|---|---|
| 17 | 23840747 | ***DNAH7*** | 0.0102 |
| 5 | 8552273 | ***PTPN4*** | 0.0109 |
| 2 | 9866197 | *RNF175* | 0.0119 |
| 2 | 5565157 | *SUCLG1* | 0.0122 |
| 28 | 8848388 | *IL1B* | 0.0124 |
| 5 | 8544538 | ***PTPN4*** | 0.0132 |
| 2 | 9865226 | *RNF175* | 0.0191 |
| 17 | 23938277 | ***DNAH7*** | 0.0234 |
| 28 | 10593199 | *NKL* | 0.0249 |
| Contig45 | 334608 | *DDX58* | 0.0274 |
| 17 | 23963829 | ***DNAH7*** | 0.0284 |
| 34 | 15833750 | *KLRF1* | 0.0288 |
| 34 | 15837599 | *KLRF1* | 0.0288 |
| 34 | 15829311 | *KLRF1* | 0.0288 |
| 34 | 15833105 | *KLRF1* | 0.0288 |
| 20 | 23100696 | ***HLA-A-24*** | 0.0294 |

d) Non-imputed dataset (first four PC)

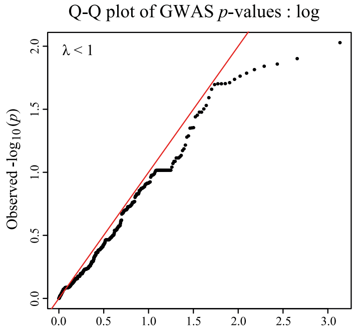


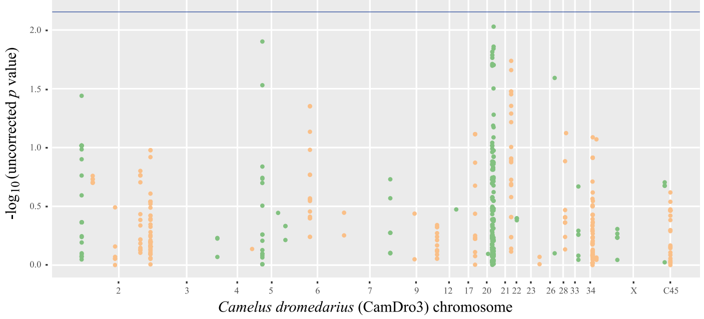


| Chr | Position | Gene | *p*-value |
|---|---|---|---|
| 20 | 23270291 | *HLA-A-30* | 0.0094 |
| 5 | 8543352 | ***PTPN4*** | 0.0125 |
| 20 | 23268649 | *HLA-A-30* | 0.0139 |
| 20 | 23101810 | ***HLA-A-24*** | 0.0144 |
| 20 | 20959362 | *RT1-Bb* | 0.0153 |
| 20 | 20959332 | *RT1-Bb* | 0.0164 |
| 20 | 20959331 | *RT1-Bb* | 0.0173 |
| 21 | 21448206 | *FCRL3* | 0.0183 |
| 20 | 20959361 | *RT1-Bb* | 0.0195 |
| 20 | 20959358 | *RT1-Bb* | 0.0198 |
| 20 | 23270314 | *HLA-A-30* | 0.0199 |
| 20 | 23270316 | *HLA-A-30* | 0.0199 |
| 20 | 20866612 | *DLA* | 0.0201 |
| 21 | 21453272 | *FCRL3* | 0.0220 |
| 26 | 17219376 | *TLR3* | 0.0256 |
| 5 | 8542959 | ***PTPN4*** | 0.0295 |

**Figure S2. Manhattan and QQ plot**. Highlighted in bold are the four genes that are common in all analyses (*HLA-A*-like, *PTPN4*, *MAGOHB* and

*DNAH7*). FDR corrected thresholds are represented in blue. a) Total dataset; b) only livestock market samples; c) only spring field samples; d) non-imputed dataset. C45 corresponds to Contig45, an unplaced scaffold.

## Supplementary tables

## Table S1. Sample information assessment of virus presence (swabs) and antibody prevalence (sera).

| No | Phenotype | Original code | Lab code | Sampling site & date | Sex (M/F) | Age (yr) | Chip ID | Farm location (UAE) | Nasal Swabs | Camel serum samples | | | Samples used in the association tests |
|---|---|---|---|---|---|---|---|---|---|---|---|---|---|
| | | | | | | | | | MERS CoV ORF1a RT-qPCR (ct value) | MERS CoV ORF1a RT-qPCR | Anti-MERS CoV ELISA (extinction ratio) | Anti-MERS-CoV-IIFT (antibody titer) | |
| **1** | V- AB- | 51 | Drom1508 | Livestock market, Oct 19 | F | 2 | 985007841357609 | Marmoom | neg. | neg. | borderline 1.1 | 1:100 | |
| **2** | V- AB- | 88 | Drom1545 | Livestock market, Oct 19 | | 6 m | NO CHIP INFO | | neg. | neg. | borderline 0.9 | 1:100 | |
| **3** | V- AB- | NSB14 | Drom1831 | Livestock market, April 19 | M | 2 | 900111881038113 | Al Saad | neg. | neg.. | borderline 1.02 | ≥ 1:1,000 | |
| **4** | V- AB- | M12 | Drom1940 | Bedouin farm, Al Mazrooei, March 19 | M | 2 m | NO CHIP INFO | | neg. | neg. | borderline 1.00 | 1:1,000 | |
| **5** | V- AB- | NSw53 | Drom1903 | Wildlife Reserve, ArabAdv, April 19 | F | 2 | NO CHIP INFO | | neg. | neg. | neg. 0.07 | neg. | |
| **6** | V- AB- | NSw54 | Drom1904 | Wildlife Reserve, ArabAdv, April 19 | M | 6 | NO CHIP INFO | | neg. | neg. | borderline 1.09 | 1:100 | |
| **7** | V- AB- | NSw57 | Drom1907 | Wildlife Reserve, DesertStar, April 19 | M | 8-9 | NO CHIP INFO | | neg. | neg. | neg. 0.64 | 1:100 | |
| **8** | V- AB+ | 13 | Drom1470 | Livestock market, Oct 19 | F | 1 | 784010050046584 | Abu Samra | doubtful 39.6 | neg. | pos. 4.0 | ≥ 1:1,000 | |
| **9** | V- AB+ | 14 | Drom1471 | Livestock market, Oct 19 | F | 6 | 992001000331305 | | neg. | neg. | pos. 3.4 | ≥ 1:1,000 | X |
| **10** | V- AB+ | 25 | Drom1482 | Livestock market, Oct 19 | F | 3 | 900057600121775 | Dubai | doubtful 40.4 | neg. | pos. 2.5 | ≥ 1:1,000 | |
| **11** | V- AB+ | 31 | Drom1488 | Livestock market, Oct 19 | M | 2 | 908182001493742 | | neg. | neg. | pos. 4.7 | ≥ 1:1,000 | X |
| **12** | V- AB+ | 35 | Drom1492 | Livestock market, Oct 19 | F | 6 | 900057600126791 | Malaghat | neg. | neg. | pos. 4.8 | ≥ 1:1,000 | X |
| **13** | V- AB+ | 37 | Drom1494 | Livestock market, Oct 19 | F | 5 | 968000002916753 | | neg. | neg. | pos. 4.5 | ≥ 1:1,000 | X |
| **14** | V- AB+ | 38 | Drom1495 | Livestock market, Oct 19 | M | 6 | 985007841229562 | Al Wagan | neg. | neg. | pos. 5.9 | ≥ 1:1,000 | X |
| **15** | V- AB+ | 39 | Drom1496 | Livestock market, Oct 19 | F | 6 | 784010050348103 | | neg. | neg. | pos. 4.1 | ≥ 1:1,000 | X |
| **16** | V- AB+ | 42 | Drom1499 | Livestock market, Oct 19 | F | 3 | 784019000006947 | Sweihan | neg. | neg. | pos. 3.5 | ≥ 1:1,000 | X |
| **17** | V- AB+ | 43 | Drom1500 | Livestock market, Oct 19 | F | 6 | 985007841219484 | Marmoom | neg. | neg. | pos. 4.8 | ≥ 1:1,000 | X |
| **18** | V- AB+ | 47 | Drom1504 | Livestock market, Oct 19 | F | 6 | 784010050073661 | Marakh | neg. | neg. | pos. 5.1 | ≥ 1:1,000 | X |

| | | | | | | | | | | | | | |
|---|---|---|---|---|---|---|---|---|---|---|---|---|---|
| **19** | V- AB+ | 48 | Drom1505 | Livestock market, Oct 19 | F | 5 | 784019000006158 | Mezyad | neg. | neg. | pos. 3.2 | 1:1,000 | X |
| **20** | V- AB+ | 69 | Drom1526 | Livestock market, Oct 19 | F | 6 | 784010050079111 | | neg. | neg. | pos. 4.6 | ≥ 1:1,000 | X |
| **21** | V- AB+ | 72 | Drom1529 | Livestock market, Oct 19 | | | 991001001739927 | | neg. | neg. | pos. 4.2 | ≥ 1:1,000 | |
| **22** | V- AB+ | 79 | Drom1536 | Livestock market, Oct 19 | F | 6 | 784010050079139 | | neg. | neg. | pos. 4.4 | ≥ 1:1,000 | X |
| **23** | V- AB+ | 85 | Drom1542 | Livestock market, Oct 19 | F | 4 | 784010050140963 | Dubai | doubtful 39.8 | neg. | pos. 3.5 | ≥ 1:1,000 | |
| **24** | V- AB+ | NSB5 | Drom1823 | Livestock market, April 19 | M | 2 | 900111881038114 | Al Saad | neg. | neg.. | pos. 2.39 | 1:1,000 | X |
| **25** | V- AB+ | NSB8 | Drom1826 | Livestock market, April 19 | M | 2 | 900111881038106 | Al Saad | neg. | neg.. | pos. 1.74 | 1:1,000 | X |
| **26** | V- AB+ | NSB12 | Drom1829 | Livestock market, April 19 | | | NO CHIP INFO | | neg. | neg.. | pos. 2.02 | 1:1,000 | |
| **27** | V- AB+ | NSB13 | Drom1830 | Livestock market, April 19 | F | 4 | 784010050423283 | Bida Bint Saud / Bad' Bint Sa'oud | neg. | neg.. | pos. 3.49 | ≥ 1:1,000 | X |
| **28** | V- AB+ | NSB16 | Drom1833 | Livestock market, April 19 | F | 4-5 | 634078000075327 | Sweihan | neg. | neg.. | pos. 3.14 | ≥ 1:1,000 | X |
| **29** | V- AB+ | NSB17 | Drom1834 | Livestock market, April 19 | F | 8 | 784010050077291 | Bida Bint Saud / Bad' Bint Sa'oud | neg. | neg.. | pos. 4.09 | ≥ 1:1,000 | X |
| **30** | V- AB+ | NSB18 | Drom1835 | Livestock market, April 19 | F | 2 | 900182001414799 | Al Jabeeb | neg. | neg.. | pos. 3.01 | ≥ 1:1,000 | X |
| **31** | V- AB+ | NSB19 | Drom1836 | Livestock market, April 19 | M | 1 | 991001002575031 | Marakh | neg. | neg.. | pos. 5.10 | ≥ 1:1,000 | X |
| **32** | V- AB+ | NSB20 | Drom1837 | Livestock market, April 19 | M | 2 | 900111880935178 | Marakh | neg. | neg.. | pos. 3.67 | ≥ 1:1,000 | X |
| **33** | V- AB+ | NSB21 | Drom1838 | Livestock market, April 19 | M | 3 | 985007841400429 | Zaid | neg. | neg.. | pos. 1.61 | ≥ 1:1,000 | X |
| **34** | V- AB+ | NSB41 | Drom1858 | Livestock market, April 19 | F | 4 | 985007841359958 | | neg. | neg.. | pos. 3.61 | 1:1,000 | X |
| **35** | V- AB+ | NSB42 | Drom1859 | Livestock market, April 19 | F | 4 | 784010050067053 | Al Kowah | neg. | neg.. | pos. 4.14 | ≥ 1:1,000 | X |
| **36** | V- AB+ | NSB46 | Drom1863 | Livestock market, April 19 | F | 6 | 991001002575819 | Mulakat | neg. | neg.. | pos. 4.46 | ≥ 1:1,000 | X |
| **37** | V- AB+ | M1 | Drom1929 | Bedouin farm, Al Mazrooei, March 19 | F | 4 | NO CHIP INFO | | neg. | neg. | pos. 3.84 | ≥ 1:1,000 | X |
| **38** | V- AB+ | M2 | Drom1930 | Bedouin farm, Al Mazrooei, March 19 | F | 4 | NO CHIP INFO | | neg. | neg. | pos. 3.96 | ≥ 1:1,000 | X |
| **39** | V- AB+ | M3 | Drom1931 | Bedouin farm, Al Mazrooei, March 19 | F | 4 | NO CHIP INFO | | neg. | neg. | pos. 2.01 | 1:1,000 | X |
| **40** | V- AB+ | M4 | Drom1932 | Bedouin farm, Al Mazrooei, March 19 | F | 15 | NO CHIP INFO | | neg. | neg. | pos. 1.35 | 1:1,000 | X |
| **41** | V- AB+ | M5 | Drom1933 | Bedouin farm, Al Mazrooei, March 19 | F | 8 | NO CHIP INFO | | neg. | neg. | pos. 4.06 | ≥ 1:1,000 | X |

| | | | | | | | | | | | | | |
|---|---|---|---|---|---|---|---|---|---|---|---|---|---|
| **42** | V- AB+ | M6 | Drom1934 | Bedouin farm, Al Mazrooei, March 19 | F | 25-30 | NO CHIP INFO | | neg. | neg. | pos. 3.89 | ≥ 1:1,000 | X |
| **43** | V- AB+ | M7 | Drom1935 | Bedouin farm, Al Mazrooei, March 19 | F | 5 | NO CHIP INFO | | neg. | neg. | pos. 4.17 | ≥ 1:1,000 | X |
| **44** | V- AB+ | M8 | Drom1936 | Bedouin farm, Al Mazrooei, March 19 | F | 12 | NO CHIP INFO | | neg. | neg. | pos. 4.03 | ≥ 1:1,000 | X |
| **45** | V- AB+ | M9 | Drom1937 | Bedouin farm, Al Mazrooei, March 19 | F | 12 | NO CHIP INFO | | neg. | neg. | pos. 2.89 | 1:1,000 | X |
| **46** | V- AB+ | M10 | Drom1938 | Bedouin farm, Al Mazrooei, March 19 | F | 12 | NO CHIP INFO | | neg. | neg. | pos. 2.96 | ≥ 1:1,000 | X |
| **47** | V- AB+ | M11 | Drom1939 | Bedouin farm, Al Mazrooei, March 19 | F | 14 | NO CHIP INFO | | neg. | neg. | pos. 2.91 | 1:1,000 | X |
| **48** | V- AB+ | M13 | Drom1941 | Bedouin farm, Al Mazrooei, March 19 | M | 10 | NO CHIP INFO | | neg. | neg. | pos. 4.04 | ≥ 1:1,000 | X |
| **49** | V- AB+ | M14 | Drom1942 | Bedouin farm, Al Mazrooei, March 19 | M | 14 | NO CHIP INFO | | neg. | neg. | pos. 4.11 | ≥ 1:1,000 | X |
| **50** | V- AB+ | M15 | Drom1943 | Bedouin farm, Al Mazrooei, March 19 | F | 10 | NO CHIP INFO | | neg. | neg. | pos. 2.90 | ≥ 1:1,000 | X |
| **51** | V- AB+ | NSw20 | Drom1872 | Wildlife Reserve, Al Maha, April 19 | M | 15 | NO CHIP INFO | | neg. | neg. | pos. 2.67 | 1:1,000 | X |
| **52** | V- AB+ | NSw21 | Drom1873 | Wildlife Reserve, Al Maha, April 19 | M | 13 | NO CHIP INFO | | neg. | neg. | pos. 3.54 | ≥ 1:1,000 | X |
| **53** | V- AB+ | NSw22 | Drom1874 | Wildlife Reserve, Al Maha, April 19 | M | 17 | NO CHIP INFO | | neg. | neg. | pos. 3.09 | 1:1,000 | X |
| **54** | V- AB+ | NSw23 | Drom1875 | Wildlife Reserve, Al Maha, April 19 | F | 14 | NO CHIP INFO | | neg. | neg. | pos. 3.40 | 1:1,000 | X |
| **55** | V- AB+ | NSw25 | Drom1877 | Wildlife Reserve, Al Maha, April 19 | M | 9 | NO CHIP INFO | | neg. | neg. | pos. 3.25 | 1:1,000 | X |
| **56** | V- AB+ | NSw26 | Drom1878 | Wildlife Reserve, Al Maha, April 19 | M | 7 | NO CHIP INFO | | neg. | neg. | pos. 2.19 | ≥ 1:1,000 | X |
| **57** | V- AB+ | NSw27 | Drom1879 | Wildlife Reserve, Al Maha, April 19 | F | 5 | NO CHIP INFO | | neg. | neg. | pos. 2.12 | 1:1,000 | X |
| **58** | V- AB+ | NSw30 | Drom1882 | Wildlife Reserve, Al Maha, April 19 | F | 14 | NO CHIP INFO | | neg. | neg. | pos. 4.26 | ≥ 1:1,000 | X |
| **59** | V- AB+ | NSw32 | Drom1884 | Wildlife Reserve, Al Maha, April 19 | M | 12 | NO CHIP INFO | | neg. | neg. | pos. 3.95 | ≥ 1:1,000 | X |
| **60** | V- AB+ | NSw34 | Drom1886 | Wildlife Reserve, Al Maha, April 19 | M | 7 | NO CHIP INFO | | neg. | neg. | pos. 3.01 | 1:100 | X |
| **61** | V- AB+ | NSw35 | Drom1887 | Wildlife Reserve, Al Maha, April 19 | M | 2 y 1 m | NO CHIP INFO | | neg. | neg. | pos. 1.87 | 1:1,000 | X |
| **62** | V- AB+ | NSw38 | Drom1890 | Wildlife Reserve, Alpha, April 19 | M | 15 | NO CHIP INFO | | neg. | neg. | pos. 3.89 | 1:1,000 | X |
| **63** | V- AB+ | NSw40 | Drom1892 | Wildlife Reserve, Travco, April 19 | M | 12 | NO CHIP INFO | | neg. | neg. | pos. 4.58 | ≥ 1:1,000 | X |
| **64** | V- AB+ | NSw42 | Drom1893 | Wildlife Reserve, Travco, April 19 | M | 9 | NO CHIP INFO | | neg. | neg. | pos. 2.86 | 1:100 | X |
| **65** | V- AB+ | NSw43 | Drom1894 | Wildlife Reserve, ArabAdv, April 19 | F | 16 | NO CHIP INFO | | neg. | neg. | pos. 4.42 | ≥ 1:1,000 | X |
| **66** | V- AB+ | NSw46 | Drom1897 | Wildlife Reserve, ArabAdv, April 19 | M | 16 | NO CHIP INFO | | neg. | neg. | pos. 3.26 | ≥ 1:1,000 | X |

| | | | | | | | | | | | | | |
|---|---|---|---|---|---|---|---|---|---|---|---|---|---|
| **67** | V- AB+ | NSw48 | Drom1899 | Wildlife Reserve, ArabAdv, April 19 | M | 12 | NO CHIP INFO | | neg. | neg. | pos. 4.55 | ≥ 1:1,000 | X |
| **68** | V- AB+ | NSw51 | Drom1901 | Wildlife Reserve, ArabAdv, April 19 | F | 16 | NO CHIP INFO | | neg. | neg. | pos. 2.76 | ≥ 1:1,000 | X |
| **69** | V- AB+ | NSw52 | Drom1902 | Wildlife Reserve, ArabAdv, April 19 | M | 11 | NO CHIP INFO | | neg. | neg. | pos. 3.24 | 1:1,000 | X |
| **70** | V- AB+ | NSw55 | Drom1905 | Wildlife Reserve, ArabAdv, April 19 | F | 18 | NO CHIP INFO | | neg. | neg. | pos. 3.37 | ≥ 1:1,000 | X |
| **71** | V- AB+ | NSw56 | Drom1906 | Wildlife Reserve, ArabAdv, April 19 | F | 6 m | NO CHIP INFO | | neg. | neg. | pos. 1.28 | 1:100 | X |
| **72** | V- AB+ | NSw59 | Drom1909 | Wildlife Reserve, DesertStar, April 19 | M | 5 | NO CHIP INFO | | neg. | neg. | pos. 2.13 | 1:1,000 | X |
| **73** | V- AB+ | NSw60 | Drom1910 | Wildlife Reserve, DesertStar, April 19 | M | 5 | NO CHIP INFO | | neg. | neg. | pos. 3.10 | 1:1,000 | X |
| **74** | V- AB+ | NSw63 | Drom1913 | Wildlife Reserve, DesertStar, April 19 | M | 15 | NO CHIP INFO | | neg. | neg. | pos. 2.35 | 1:1,000 | X |
| **75** | V- AB+ | NSw67 | Drom1917 | Wildlife Reserve, DesertStar, April 19 | M | 18 | NO CHIP INFO | | neg. | neg. | pos. 3.02 | 1:1,000 | X |
| **76** | V- AB+ | NSw69 | Drom1919 | Wildlife Reserve, DesertStar, April 19 | M | 18 | NO CHIP INFO | | neg. | neg. | pos. 3.34 | ≥ 1:1,000 | X |
| **77** | V- AB+ | NSw79 | Drom1928 | Wildlife Reserve, DesertStar, April 19 | M | 7 | NO CHIP INFO | | neg. | neg. | pos. 3.42 | 1:1,000 | X |
| **78** | V+ AB- | 2 | Drom1459 | Livestock market, Oct 19 | M | 4 m | NO CHIP INFO | | pos. 29.3 | neg. | neg. 0.1 | neg. | |
| **79** | V+ AB- | 5 | Drom1462 | Livestock market, Oct 19 | M | 1 | 900057600126219 | Marakh | pos. 19.9 | neg. | neg. 0.1 | neg. | |
| **80** | V+ AB- | 6 | Drom1463 | Livestock market, Oct 19 | M | 3 m | NO CHIP INFO | | pos. 25.1 | neg. | neg. 0.1 | neg. | |
| **81** | V+ AB- | 7 | Drom1464 | Livestock market, Oct 19 | M | 2 m | NO CHIP INFO | | pos. 34.2 | neg. | neg. 0.5 | 1:100 | |
| **82** | V+ AB- | 54 | Drom1511 | Livestock market, Oct 19 | M | 2 | 900057600121585 | | pos. 33.4 | neg. | borderline 0.9 | 1:1,000 | |
| **83** | V+ AB- | 87 | Drom1544 | Livestock market, Oct 19 | M | 1 | 900057600121595 | | Pos. 39.2 | neg. | neg. 0.3 | 1:100 | |
| **84** | V+ AB- | NSB40 | Drom1857 | Livestock market, April 19 | | 2 m | NO CHIP INFO | | pos. 26.1 | neg.. | neg. 0.33 | 1:100 | |
| **85** | V+ AB+ | 1 | Drom1458 | Livestock market, Oct 19 | M | 2 | 900182001697895 | | pos. 34.8 | neg. | pos. 3.6 | ≥ 1:1,000 | X |
| **86** | V+ AB+ | 3 | Drom1460 | Livestock market, Oct 19 | M | 2 | 900215000003532 | | pos. 30.3 | neg. | pos. 4.1 | ≥ 1:1,000 | X |
| **87** | V+ AB+ | 4 | Drom1461 | Livestock market, Oct 19 | | | 900111881027297 | | pos. 36.7 | neg. | pos. 1.9 | 1:1,000 | |
| **88** | V+ AB+ | 8 | Drom1465 | Livestock market, Oct 19 | F | 3 | 900057600122200 | Abu Dhabi | pos. 36.2 | neg. | pos. 5.0 | ≥ 1:1,000 | X |
| **89** | V+ AB+ | 9 | Drom1466 | Livestock market, Oct 19 | F | 3 | 900215000005008 | Abu Dhabi | pos. 36.8 | neg. | pos. 5.3 | ≥ 1:1,000 | X |
| **90** | V+ AB+ | 10 | Drom1467 | Livestock market, Oct 19 | F | 5 | 985007841277728 | Marakh | pos. 37.1 | neg. | pos. 2.6 | ≥ 1:1,000 | X |
| **91** | V+ AB+ | 11 | Drom1468 | Livestock market, Oct 19 | F | 6 | 784010050031772 | Mulakat | pos. 36.3 | neg. | pos. 4.8 | ≥ 1:1,000 | X |

| | | | | | | | | | | | | | | |
|---|---|---|---|---|---|---|---|---|---|---|---|---|---|---|
| **92** | V+ AB+ | 23 | Drom1480 | Livestock market, Oct 19 | F | 6 | 784010050267117 | | pos. 36.9 | neg. | pos. 4.5 | ≥ 1:1,000 | X |
| **93** | V+ AB+ | 27 | Drom1484 | Livestock market, Oct 19 | F | 6m | 784010050465212 | Al Yahar | pos. 35.1 | neg. | pos. 5.0 | ≥ 1:1,000 | X |
| **94** | V+ AB+ | 28 | Drom1485 | Livestock market, Oct 19 | M | 5 m | NO CHIP INFO | | pos. 27.2 | neg. | pos. 4.5 | ≥ 1:1,000 | X |
| **95** | V+ AB+ | 29 | Drom1486 | Livestock market, Oct 19 | M | 6 m | NO CHIP INFO | | pos. 35.4 | neg. | pos. 1.8 | ≥ 1:1,000 | X |
| **96** | V+ AB+ | 32 | Drom1489 | Livestock market, Oct 19 | M | 2 | 991001002574519 | | pos. 38.6 | neg. | pos. 4.0 | ≥ 1:1,000 | X |
| **97** | V+ AB+ | 45 | Drom1502 | Livestock market, Oct 19 | M | 5 | 985007841209387 | Khushaba | pos. 38.0 | neg. | pos. 4.6 | ≥ 1:1,000 | X |
| **98** | V+ AB+ | 56 | Drom1513 | Livestock market, Oct 19 | F | 2 | 784010050551133 | Jabib | pos. 36.1 | neg. | pos. 4.2 | ≥ 1:1,000 | X |
| **99** | V+ AB+ | 90 | Drom1547 | Livestock market, Oct 19 | F | 6 | 784010050294118 | Marakh | pos. 35.8 | neg. | pos. 4.3 | ≥ 1:1,000 | X |
| **100** | V+ AB+ | NSB10 | Drom1827 | Livestock market, April 19 | F | 2 | 784010050550508 | Seih Sabra/Sih Sabra/Seeh Sabra | pos. 38.2 | neg. | pos. 1.87 | 1:1,000 | X |
| **101** | V+ AB+ | NSB11 | Drom1828 | Livestock market, April 19 | M | 2 | 784010050550691 | Mezyad | pos. 34.9 | neg. | pos. 2.73 | 1:1,000 | X |
| **102** | V+ AB+ | NSB23 | Drom1840 | Livestock market, April 19 | M | 1 | 991001002570111 | | pos. 32.6 | neg. | pos. 4.34 | ≥ 1:1,000 | X |
| **103** | V+ AB+ | NSB30 | Drom1847 | Livestock market, April 19 | F | 4 | 784010050229794 | Badr Zaid | pos. 39.5 | neg. | pos. 1.96 | 1:1,000 | X |
| **104** | V+ AB+ | NSB31 | Drom1848 | Livestock market, April 19 | F | 6 | 784010050243508 | | pos. 39.0 | neg. | pos. 4.69 | ≥ 1:1,000 | X |
| **105** | V+ AB+ | NSB33 | Drom1850 | Livestock market, April 19 | M | 1-2 | 992001000330620 | Malaqāt | pos. 32.3 | neg. | pos. 4.67 | ≥ 1:1,000 | X |
| **106** | V+ AB+ | NSB34 | Drom1851 | Livestock market, April 19 | F | 2 | 991001002575035 | | pos. 35.1 | neg. | pos. 4.44 | 1:1,000 | X |
| **107** | V+ AB+ | NSB35 | Drom1852 | Livestock market, April 19 | F | 3 | 991001002574462 | Sweihan | pos. 33.4 | neg. | pos. 1.37 | 1:100 | X |
| **108** | V+ AB+ | NSB36 | Drom1853 | Livestock market, April 19 | M | 3 | 991001002575745 | Jebayeb | pos. 35.4 | neg. | pos. 1.33 | 1:100 | X |
| **109** | V+ AB+ | NSB37 | Drom1854 | Livestock market, April 19 | M | 2-3 | 992001000330472 | Al Saad | pos. 34.1 | neg. | pos. 1.84 | 1:100 | X |
| **110** | V+ AB+ | NSB38 | Drom1855 | Livestock market, April 19 | M | 3 | 991001002575920 | | pos. 37.5 | neg. | pos. 4.02 | ≥ 1:1,000 | X |
| **111** | V+ AB+ | NSB39 | Drom1856 | Livestock market, April 19 | F | 6 | 784010050365484 | RAK | pos. 33.5 | neg. | pos. 4.67 | ≥ 1:1,000 | X |
| **112** | V+ AB+ | NSB43 | Drom1860 | Livestock market, April 19 | M | 1-2 | 992001000330719 | Marakh | pos. 37.4 | neg. | pos. 2.40 | 1:100 | X |
| **113** | V+ AB+ | NSB44 | Drom1861 | Livestock market, April 19 | F | 6 | 784010050516433 | | pos. 37.5 | neg. | pos. 4.82 | ≥ 1:1,000 | X |
| **114** | V+ AB+ | NSB45 | Drom1862 | Livestock market, April 19 | F | 3 | 784010050028578 | Jabib – Al Faqa | pos. 38.9 | neg. | pos. 3.53 | ≥ 1:1,000 | X |
| **115** | V+ AB+ | NSB47 | Drom1864 | Livestock market, April 19 | M | 3 | 991001002575899 | Marmoon | pos. 35.2 | neg. | pos. 1.76 | 1:1,000 | X |
| **116** | V+ AB+ | NSB48 | Drom1865 | Livestock market, April 19 | M | 1-2 | 990001000053606 | Sweihan | pos. 33.7 | neg. | pos. 4.82 | ≥ 1:1,000 | X |

| | | | | | | | | | | | | | |
|---|---|---|---|---|---|---|---|---|---|---|---|---|---|
| **117** | V+ AB+ | NSB49 | Drom1866 | Livestock market, April 19 | F | 3 | 900111881038306 | | pos. 36.1 | neg. | pos. 5.47 | ≥ 1:1,000 | X |
| **118** | V+ AB+ | NSB50 | Drom1867 | Livestock market, April 19 | | 2m | NO CHIP INFO | | pos. 38.5 | neg. | pos. 3.17 | 1:1,000 | X |
| **119** | V+ AB+ | NSB51 | Drom1868 | Livestock market, April 19 | M | 2 | 991001002574752 | RAK | pos. 30.0 | neg. | pos. 2.04 | 1:1,000 | X |
| **120** | V+ AB+ | NSB52 | Drom1869 | Livestock market, April 19 | M | 1-2 | 900074001585559 | Shabiah | pos. 39.3 | neg. | pos. 4.91 | ≥ 1:1,000 | X |
| **121** | V+ AB+ | NSB54 | Drom1871 | Livestock market, April 19 | F | 5 | 784010050359111 | | pos. 36.3 | neg. | pos. 3.89 | 1:1,000 | X |

**Table S2. Scheme showing how IIFT antibody titers were determined according to the fluorescence of the different sample dilutions.**

| **Sample dilutions / Fluorescent signal** | | | **Antibody Titer** |
|---|---|---|---|
| **1:10** | **1:100** | **1:1,000** | |
| weak | negative | negative | **1:10** |
| moderate | negative | negative | **1:10** |
| strong | weak | negative | **1:100** |
| strong | moderate | negative | **1:100** |
| strong | strong | weak | **1:1,000** |
| strong | strong | moderate | **≥ 1:1,000** |
| strong | strong | strong | **≥ 1:1,000** |

**Table S3. Observed ($H_O$) and expected ($H_E$) heterozygosity values depicted in immune response gene groups.** Identified candidate genes *MAGOHB*, *HLA-A-24*-like, *HLA-DPB1*-like, *DNAH7* and *PTPN4* are highlighted in bold.

| Gene ID | Genes-No.SNPs | Genes-$H_O$ | Genes-$H_E$ | Exons-No.SNPs | Exons-$H_O$ | Exons-$H_E$ | Introns-No.SNPs | Introns-$H_O$ | Introns-$H_E$ | Name | Description |
|---|---|---|---|---|---|---|---|---|---|---|---|
| **Granzyme** | | | | | | | | | | | |
| Cadr_00004168 | 4 | 0.272 | 0.329 | 0 | NA | NA | 4 | 0.272 | 0.329 | *GZMA* | Granzyme A (Bos taurus OX=9913) |
| Cadr_00004169 | 1 | NA | NA | 0 | NA | NA | 1 | NA | NA | *GZMA* | Granzyme A (Homo sapiens OX=9606) |
| Cadr_00005822 | 0 | NA | NA | 0 | NA | NA | 0 | NA | NA | *GZMB* | Granzyme B (Homo sapiens OX=9606) |
| Cadr_00005823 | 22 | 0.24 | 0.246 | 1 | NA | NA | 22 | 0.24 | 0.246 | *GZMB* | Granzyme B (Homo sapiens OX=9606) |
| Cadr_00005821 | 5 | 0.411 | 0.38 | 0 | NA | NA | 5 | 0.411 | 0.38 | *GZMH* | Granzyme H (Homo sapiens OX=9606) |
| Cadr_00004167 | 6 | 0.29 | 0.343 | 1 | NA | NA | 5 | 0.27 | 0.32 | *GZMK* | Granzyme K (Homo sapiens OX=9606) |
| Cadr_00025032 | 16 | 0.246 | 0.255 | 3 | 0.056 | 0.066 | 13 | 0.289 | 0.299 | *GZMM* | Granzyme M (Homo sapiens OX=9606) |
| **Mean** | | **0.29** | **0.31** | | **0.06** | **0.07** | | **0.30** | **0.31** | | |
| **Interleukin** | | | | | | | | | | | |
| Cadr_00001885 | 3 | 0.423 | 0.484 | 0 | NA | NA | 3 | 0.423 | 0.484 | *CXCL8* | Interleukin-8 (Canis lupus familiaris OX=9615) |
| Cadr_00023412 | 7 | 0.197 | 0.197 | 2 | 0.158 | 0.146 | 5 | 0.213 | 0.217 | *IL10* | Interleukin-10 (Lama glama OX=9844) |
| Cadr_00028914 | 14 | 0.201 | 0.228 | 11 | 0.187 | 0.22 | 9 | 0.246 | 0.278 | *IL10RA* | Interleukin-10 receptor subunit alpha (Homo sapiens OX=9606) |
| Cadr_00001098 | 61 | 0.165 | 0.168 | 1 | NA | NA | 61 | 0.165 | 0.168 | *IL10RB* | Interleukin-10 receptor subunit alpha (Homo sapiens OX=9606) |
| Cadr_00029940 | 16 | 0.32 | 0.318 | 5 | 0.19 | 0.188 | 11 | 0.379 | 0.377 | *IL1A* | Interleukin-1 alpha (Lama glama OX=9844) |
| Cadr_00029941 | 20 | 0.285 | 0.308 | 2 | 0.306 | 0.317 | 18 | 0.283 | 0.307 | *IL1B* | Interleukin-1 beta (Lama glama OX=9844) |
| **Mean** | | **0.27** | **0.28** | | **0.21** | **0.22** | | **0.28** | **0.31** | | |
| **Killer cell** | | | | | | | | | | | |
| Cadr_00029273 | 19 | 0.277 | 0.295 | 3 | 0.169 | 0.179 | 16 | 0.297 | 0.317 | *Klra2* | Killer cell lectin-like receptor 2 (Mus musculus OX=10090) |
| Cadr_00029303 | 4 | 0.229 | 0.22 | 0 | NA | NA | 4 | 0.229 | 0.22 | *KLRB1* | Killer cell lectin-like receptor subfamily B member 1 (Homo sapiens OX=9606) |
| Cadr_00029300 | 2 | 0.448 | 0.399 | 2 | 0.448 | 0.399 | 0 | NA | NA | *Klrb1b* | Killer cell lectin-like receptor subfamily B member 1B allele A (Camelus bactrianus XP_010944886.1) |
| Cadr_00029489 | 18 | 0.348 | 0.389 | 1 | NA | NA | 17 | 0.344 | 0.382 | *KLRC2* | NKG2-C type II integral membrane protein (Homo sapiens OX= 9606) |
| Cadr_00029281 | 10 | 0.315 | 0.349 | 8 | 0.289 | 0.323 | 7 | 0.312 | 0.34 | *KLRD1* | Natural killer cells antigen CD94 (Bos taurus OX=9913) |
| Cadr_00029283 | 13 | 0.345 | 0.35 | 1 | NA | NA | 12 | 0.341 | 0.347 | *Klre1* | Killer cell lectin-like receptor subfamily E member 1 (Mus musculus OX=10090) |
| Cadr_00029297 | 73 | 0.458 | 0.435 | 3 | 0.374 | 0.367 | 70 | 0.462 | 0.438 | *KLRF1* | Killer cell lectin-like receptor subfamily F member 1 (Macaca fascicularis OX=9541) |
| Cadr_00029295 | 48 | 0.266 | 0.244 | 3 | 0.294 | 0.261 | 47 | 0.266 | 0.244 | *KLRF2* | Killer cell lectin-like receptor subfamily F member 2 (Homo sapiens OX=9606) |
| Cadr_00008447 | 42 | 0.352 | 0.354 | 11 | 0.414 | 0.411 | 31 | 0.33 | 0.334 | *Klrg2* | Killer cell lectin-like receptor subfamily G member 2 (Mus musculus OX=10090) |
| Cadr_00029277 | 28 | 0.369 | 0.403 | 1 | NA | NA | 28 | 0.369 | 0.403 | *Klri1* | Killer cell lectin-like receptor subfamily I member 1 (Mus musculus OX=10090) |
| Cadr_00029276 | 11 | 0.406 | 0.419 | 1 | NA | NA | 11 | 0.406 | 0.419 | *KLRK1* | NKG2-D type II integral membrane protein (Pongo Pygmaeus OX=9600) |
| Cadr_00029279 | 14 | 0.244 | 0.251 | 4 | 0.248 | 0.254 | 10 | 0.242 | 0.249 | *KLRK1* | NKG2-D type II integral membrane protein (Sus scrofa OX=9823) |
| **Mean** | | **0.34** | **0.34** | | **0.32** | **0.31** | | **0.33** | **0.34** | | |
| **MHC Class I** | | | | | | | | | | | |
| Cadr_00022140 | 112 | 0.09 | 0.116 | 15 | 0.061 | 0.067 | 97 | 0.094 | 0.124 | ***HLA-A-24*-like** | HLA class I histocompatibility antigen, A-24 alpha chain (Homo sapiens OX=9606) |

| | | | | | | | | | | | |
|---|---|---|---|---|---|---|---|---|---|---|---|
| Cadr_00022145 | 30 | 0.276 | 0.273 | 8 | 0.318 | 0.322 | 22 | 0.261 | 0.256 | *HLA-A-11* | HLA class I histocompatibility antigen, A-11 alpha chain (Homo sapiens OX=9606) |
| Cadr_00022149 | 14 | 0.216 | 0.279 | 12 | 0.22 | 0.285 | 5 | 0.181 | 0.215 | *HLA-A-69* | HLA class I histocompatibility antigen, A-69 alpha chain (Homo sapiens OX=9606) |
| Cadr_00022150 | 134 | 0.191 | 0.263 | 29 | 0.196 | 0.288 | 116 | 0.191 | 0.261 | *HLA-A-30* | HLA class I histocompatibility antigen, A-30 alpha chain (Homo sapiens OX=9606) |
| Cadr_00022148 | 20 | 0.225 | 0.329 | 10 | 0.21 | 0.301 | 10 | 0.241 | 0.357 | *HLA-C* | HLA class I histocompatibility antigen, Cw-6 alpha chain (Homo sapiens OX=9606) |
| Cadr_00022156 | 4 | 0.028 | 0.038 | 2 | 0.026 | 0.045 | 2 | 0.031 | 0.03 | *Patr* | class I histocompatability B-1 alpha chain (Fragment) (Pan troglodytes OX=9598) |
| Cadr_00022105 | 18 | 0.197 | 0.187 | 10 | 0.203 | 0.188 | 8 | 0.191 | 0.184 | *Patr-A* | Patr class I histocompatibility antigen, A-126 alpha chain (Pan troglodytes OX=9598) |
| Cadr_00022139 | 67 | 0.315 | 0.318 | 3 | 0.239 | 0.241 | 64 | 0.318 | 0.322 | *Patr-A* | Patr class I histocompatibility antigen, A-126 alpha chain (Pan troglodytes OX=9598) |
| Cadr_00022147 | 5 | 0.02 | 0.032 | 4 | 0.025 | 0.035 | 1 | NA | NA | *Patr-A* | Patr class I histocompatibility antigen, A-126 alpha chain (Pan troglodytes OX=9598) |
| Cadr_00022160 | 31 | 0.047 | 0.09 | 3 | 0.048 | 0.075 | 28 | 0.047 | 0.092 | *Patr-A* | Patr class I histocompatibility antigen, A-126 alpha chain (Pan troglodytes OX=9598) |
| Cadr_00022155 | 0 | NA | NA | 0 | NA | NA | 0 | NA | NA | *Popy* | class I histocompatability antigen A-1 alpha chain (Pongo pygmaeus OX=9600) |
| **Mean** | | **0.16** | **0.19** | | **0.15** | **0.18** | | **0.17** | **0.20** | | |
| **MHC Class II** | | | | | | | | | | | |
| Cadr_00022027 | 28 | 0.35 | 0.343 | 2 | 0.48 | 0.45 | 26 | 0.339 | 0.334 | *BoLA-DQB* | BoLa class II histocompatability antigen, DQB*0101 beta chain (Bos taurus OX=9913) |
| Cadr_00004894 | 11 | 0.324 | 0.299 | 2 | 0.397 | 0.378 | 9 | 0.307 | 0.281 | *CD74* | HLA class II histocompatability antigen gamma chain (Homo sapiens OX=9606) |
| Cadr_00022030 | 95 | 0.412 | 0.402 | 37 | 0.437 | 0.416 | 58 | 0.396 | 0.393 | *DLA* | class II histocompatability antigen, DR-1 beta chain (Canis lupus familiaris OX=9615) |
| Cadr_00022020 | 5 | 0.27 | 0.307 | 2 | 0.21 | 0.245 | 3 | 0.31 | 0.348 | *HLA-DMA* | HLA class II histocompatability antigen, DM alpha chain (Homo sapiens OX=9606) |
| Cadr_00022021 | 33 | 0.287 | 0.319 | 8 | 0.292 | 0.335 | 25 | 0.285 | 0.314 | *HLA-DMB* | HLA class II histocompatability antigen, DM alpha chain (Homo sapiens OX=9606) |
| Cadr_00022018 | 25 | 0.27 | 0.295 | 17 | 0.268 | 0.293 | 8 | 0.274 | 0.3 | *HLA-DOA* | HLA class II histocompatability antigen, DO alpha chain (Homo sapiens OX=9606) |
| Cadr_00022026 | 72 | 0.328 | 0.328 | 10 | 0.245 | 0.267 | 62 | 0.342 | 0.338 | *HLA-DOB* | HLA class II histocompatability antigen, DO alpha chain (Homo sapiens OX=9606) |
| Cadr_00022017 | 32 | 0.274 | 0.296 | 5 | 0.253 | 0.274 | 27 | 0.278 | 0.3 | *HLA-DPA1* | HLA class II histocompatability antigen, DP alpha chain (Homo sapiens OX=9606) |
| Cadr_00022016 | 41 | 0.273 | 0.331 | 9 | 0.253 | 0.307 | 32 | 0.279 | 0.338 | ***HLA-DPB1-*** **like** | HLA class II histocompatability antigen, DP alpha chain (Homo sapiens OX=9606) |
| Cadr_00022036 | 89 | 0.304 | 0.372 | 13 | 0.245 | 0.386 | 76 | 0.314 | 0.369 | *HLA-DRB1* | HLA class II histocompatability antigen, DRB1-4 alpha chain (Homo sapiens OX=9606) |
| Cadr_00022037 | 63 | 0.414 | 0.43 | 6 | 0.32 | 0.318 | 57 | 0.424 | 0.442 | *HLA-DRB1* | HLA class II histocompatability antigen, DRB1-1 alpha chain (Homo sapiens OX=9606) |
| Cadr_00022038 | 34 | 0.219 | 0.236 | 13 | 0.241 | 0.26 | 21 | 0.206 | 0.221 | *Mamu-DRA* | Mamu class II histocompatibility antigen, DR alpha chain (Macac mulata OX=9544) |
| Cadr_00022032 | 7 | 0.048 | 0.101 | 2 | 0 | 0.022 | 5 | 0.067 | 0.133 | *RT1-Bb* | Rano class II histocompatibility antigen, B-1 beta chain (Rattus norvegicus OX=10116) |
| Cadr_00022034 | 36 | 0.172 | 0.202 | 2 | 0.304 | 0.266 | 34 | 0.164 | 0.198 | *RT1-Bb* | Rano class II histocompatibility antigen, B-1 beta chain (Rattus norvegicus OX=10116) |
| Cadr_00022028 | 15 | 0.423 | 0.378 | 9 | 0.426 | 0.383 | 6 | 0.417 | 0.372 | *SLA* | class II histocompatibility antigem, DQ haplotype D alpha chain (Sus scrofa =X=9823) |
| Cadr_00022033 | 6 | 0.003 | 0.023 | 0 | NA | NA | 6 | 0.003 | 0.023 | *SLA* | class II histocompatibility antigem, DQ haplotype D alpha chain (Sus scrofa =X=9823) |
| Cadr_00022035 | 4 | 0.012 | 0.047 | 3 | 0.013 | 0.046 | 1 | NA | NA | *SLA* | class II histocompatibility antigem, DQ haplotype D alpha chain (Sus scrofa =X=9823) |
| **Mean** | | **0.26** | **0.28** | | **0.27** | **0.29** | | **0.28** | **0.29** | | |
| **TLR** | | | | | | | | | | | |
| Cadr_00002152 | 5 | 0.192 | 0.19 | 5 | 0.192 | 0.19 | 0 | NA | NA | *TLR1* | Toll-like receptor 1 (Homo sapiens OX=9606) |
| Cadr_00002153 | 23 | 0.274 | 0.304 | 16 | 0.291 | 0.317 | 9 | 0.222 | 0.256 | *TLR10* | Toll-like receptor 10 (Bos taurus OX=9913) |
| Cadr_00001385 | 10 | 0.398 | 0.437 | 10 | 0.398 | 0.437 | 3 | 0.355 | 0.363 | *TLR2* | Toll-like receptor 2 (Equus caballus OX=9796) |
| Cadr_00026583 | 31 | 0.384 | 0.398 | 6 | 0.24 | 0.253 | 25 | 0.419 | 0.433 | *TLR3* | Toll-like receptor 3 (Boselaphus tragocamelus OX=9917) |
| Cadr_00016120 | 8 | 0.222 | 0.21 | 1 | NA | NA | 7 | 0.224 | 0.213 | *TLR4* | Toll-like receptor 4 (Sus scrofa OX=9823) |
| Cadr_00023195 | 4 | 0.453 | 0.46 | 4 | 0.453 | 0.46 | 0 | NA | NA | *TLR5* | Toll-like receptor 5 (Homo sapiens OX=9606) |
| Cadr_00002151 | 15 | 0.195 | 0.217 | 2 | 0.112 | 0.124 | 13 | 0.208 | 0.231 | *TLR6* | Toll-like receptor 6 (Homo sapiens OX=9606) |
| Cadr_00003728 | 18 | 0.186 | 0.376 | 1 | NA | NA | 17 | 0.186 | 0.37 | *TLR7* | Toll-like receptor 7 (Homo sapiens OX=9606) |
| Cadr_00003726 | 7 | 0.166 | 0.302 | 7 | 0.166 | 0.302 | 0 | NA | NA | *TLR8* | Toll-like receptor 8 (Homo sapiens OX=9606) |
| Cadr_00020415 | 3 | 0.088 | 0.083 | 3 | 0.088 | 0.083 | 0 | NA | NA | *TLR9* | Toll-like receptor 9 (Sus scrofa OX=9823) |

| | | | | | | | | | | | |
|---|---|---|---|---|---|---|---|---|---|---|---|
| **Other IR** | | | | | | | | | | | |
| Cadr_00030052 | 11 | 0.278 | 0.295 | 11 | 0.278 | 0.295 | 7 | 0.293 | 0.309 | *ACO1* | Cytoplasmic aconitate hidratase (Bos taurus OX=9913) |
| Cadr_00020478 | 5 | 0.127 | 0.135 | 5 | 0.127 | 0.135 | 5 | 0.127 | 0.135 | *APPL1* | DCC-interacting protein 13-alpha (Homo sapiens OX=9606) |
| Cadr_00002239 | 297 | 0.17 | 0.171 | 66 | 0.248 | 0.251 | 239 | 0.149 | 0.15 | *CC2D2A* | Coiled-coil and C2 domain-containing protein 2A (Homo sapiens OX=9606) |
| Cadr_00029296 | 27 | 0.335 | 0.316 | 2 | 0.319 | 0.284 | 27 | 0.335 | 0.316 | *CLEC2B* | C-type lectin domain family 2 member B (Homo sapiens OX=9606) |
| Cadr_00007342 | 11 | 0.226 | 0.215 | 4 | 0.282 | 0.267 | 7 | 0.194 | 0.184 | *CXCR2* | C-X-C chemokine receptor type 2 (Bos taurus OX=9913) |
| Cadr_00030053 | 90 | 0.284 | 0.312 | 11 | 0.278 | 0.295 | 86 | 0.286 | 0.314 | *DDX58* | Probable ATP-dependent RNA helicase DDX58 (Sus scrofa OX=9823) |
| Cadr_00020479 | 129 | 0.269 | 0.28 | 15 | 0.251 | 0.261 | 120 | 0.264 | 0.276 | ***DNAH7*** | Dynein heavy chain 7 axonemal (Homo sapiens OX=9607) |
| Cadr_00006877 | 64 | 0.169 | 0.176 | 1 | NA | NA | 63 | 0.169 | 0.176 | *DPP4* | Dipeptidyl peptidase 4 (Bos taurus OX=9913) |
| Cadr_00012213 | 12 | 0.352 | 0.352 | 4 | 0.425 | 0.443 | 8 | 0.316 | 0.307 | *FCAR* | Immunoglobulin alpha Fc receptor (Homo sapiens OX=9606) |
| Cadr_00024638 | 47 | 0.292 | 0.306 | 16 | 0.319 | 0.343 | 36 | 0.296 | 0.31 | *FCRL3* | Fc receptor-like protein 3 (Homo sapiens OX=9606) |
| Cadr_00011189 | 2 | 0.291 | 0.275 | 1 | NA | NA | 1 | NA | NA | *HP* | Haptoglobin (Sus scrofa OX=9823) |
| Cadr_00006880 | 29 | 0.17 | 0.201 | 1 | NA | NA | 28 | 0.162 | 0.193 | *IFIH1* | Interferon-induced helicase C domain-containing protein 1 (Homo sapiens OX=9606) |
| Cadr_00015578 | 0 | NA | NA | 0 | NA | NA | 0 | NA | NA | *IFNB2* | Interferon beta-2 (Bos taurus OX=9913) |
| Cadr_00017035 | 3 | 0.177 | 0.205 | 0 | NA | NA | 3 | 0.177 | 0.205 | *IFNG* | Interferon gamma (Camelus bactrianus OX=9837) |
| Cadr_00001103 | 50 | 0.279 | 0.258 | 0 | NA | NA | 50 | 0.279 | 0.258 | *IFNGR2* | Interferon gamma receptor 2 (Homo sapiens OX=9606) |
| Cadr_00029272 | 12 | 0.26 | 0.279 | 4 | 0.126 | 0.129 | 8 | 0.327 | 0.355 | ***MAGOHB*** | Protein mago nashi homolog 2 (Bos taurus OX=9913) |
| Cadr_00004186 | 13 | 0.241 | 0.256 | 3 | 0.293 | 0.316 | 10 | 0.226 | 0.238 | *MAP3K1* | Mitogen-activated protein kinase kinase kinase 1 (Homo sapiens OX=9606) |
| Cadr_00005819 | 1 | NA | NA | 0 | NA | NA | 1 | NA | NA | *Mast* | cell protease 3 (Ovis aries OX=9940) |
| Cadr_00012215 | 15 | 0.306 | 0.354 | 3 | 0.334 | 0.391 | 12 | 0.299 | 0.345 | *NCR1* | Natural cytoxicity triggering receptor 1 (Bos taurus OX=9913) |
| Cadr_00021869 | 9 | 0.174 | 0.186 | 3 | 0.099 | 0.095 | 6 | 0.212 | 0.232 | *NCR2* | Natural cytoxicity triggering receptor 2 (Homo sapiens OX=9606) |
| Cadr_00001692 | 77 | 0.266 | 0.254 | 2 | 0.243 | 0.248 | 75 | 0.267 | 0.254 | *NFKB1* | Nuclear factor NF-kappa-B p105 subunit (Canis lupus familiaris OX=9615) |
| Cadr_00009474 | 6 | 0.273 | 0.27 | 2 | 0.352 | 0.34 | 4 | 0.234 | 0.235 | *NFKB2* | Nuclear factor NF-kappa-B p100 subunit (Homo sapiens OX=9606) |
| Cadr_00029278 | 15 | 0.426 | 0.47 | 2 | 0.45 | 0.503 | 13 | 0.422 | 0.465 | *NKG2A* | NKG2-A/NKG2-B type II integral membrane protein (Macaca mulatta OX=9544) |
| Cadr_00029993 | 12 | 0.087 | 0.098 | 1 | NA | NA | 11 | 0.093 | 0.102 | *NKL* | Antimicrobial peptide NK-lysin (Fragment) (Sus scrofa OX=9823) |
| Cadr_00009139 | 6 | 0.371 | 0.378 | 5 | 0.348 | 0.357 | 1 | NA | NA | *PRF1* | Perforin-1 (Homo sapiens OX=9606) |
| Cadr_00007027 | 22 | 0.243 | 0.239 | 3 | 0.272 | 0.273 | 20 | 0.244 | 0.238 | *PRKRA* | Interferon-inducible double-stranded RNA_dependent protein kinase activator A (Homo sapiens OX=9606) |
| Cadr_00009475 | 0 | NA | NA | 0 | NA | NA | 0 | NA | NA | *Psd* | PH and SEC7 domain-containing protein 1 (Mus musculus OX=10090) |
| Cadr_00024639 | 2 | 0.399 | 0.432 | 2 | 0.399 | 0.432 | 2 | 0.399 | 0.432 | *PTMA* | Prothymosin alpha (Pongo abelii OX=9601) |
| Cadr_00006681 | 163 | 0.213 | 0.222 | 17 | 0.299 | 0.318 | 153 | 0.207 | 0.215 | ***PTPN4*** | Tyrosine-protein phosphatase non-receptor type 4 (Homo sapiens OX=9606) |
| Cadr_00001384 | 10 | 0.398 | 0.437 | 10 | 0.398 | 0.437 | 3 | 0.355 | 0.363 | *RNF175* | RING finger protein 175 (Homo sapiens OX=9606) |
| Cadr_00004895 | 4 | 0.333 | 0.3 | 0 | NA | NA | 4 | 0.333 | 0.3 | *Rps14* | 40S ribosomal protein S14 (Mus nusculus OX=10090) |
| Cadr_00017710 | 6 | 0.41 | 0.45 | 2 | 0.335 | 0.386 | 4 | 0.448 | 0.481 | *Rps7* | 40S ribosomal protein S7 (Rattus norvegicus OX=10116) |
| Cadr_00001327 | 32 | 0.275 | 0.273 | 14 | 0.26 | 0.259 | 18 | 0.287 | 0.284 | *Suclg1* | Succinate--CoA ligase [ADP/GDP-forming] subunit alpha, mitochondrial (Mus musculus OX=10090) |
| Cadr_00002785 | 18 | 0.132 | 0.265 | 1 | NA | NA | 17 | 0.131 | 0.261 | *Tmem255a* | Transmembrane protein 255A (Mus musculus OX=10090) |
| Cadr_00022101 | 7 | 0.188 | 0.178 | 4 | 0.226 | 0.211 | 3 | 0.138 | 0.134 | *TNF* | Tumor necrosis factor (Camelus bactrianus OX=9837 |
| Cadr_00006503 | 45 | 0.372 | 0.4 | 19 | 0.365 | 0.393 | 29 | 0.377 | 0.407 | *Traf3* | TNF receptor-associated factor 3 (Mus musculus OX=10090) |
| Cadr_00011190 | 1 | NA | NA | 1 | NA | NA | 0 | NA | NA | *TXNL4B* | Thioredoxin-like protein 4B (Homo sapiens OX=9606) |
| **Mean** | | **0.27** | **0.28** | | **0.29** | **0.31** | | **0.26** | **0.27** | | |

**Table S4. Read-based imputation performance**. nAncestralHaplotypes (k) = number of ancestral haplotypes; nGen = number of generations ago, controls recombination rate; nDiff_from_non-imputed = number of genotypes that were not the same between imputed and non-imputed samples; nMatch_from_non-imputed = number of genotypes that were the same between imputed and non-imputed sample; nMissing_from_non-imputed = these are SNPs and hence genotypes that are missing because that SNP failed QC for imputation; nAdditional_SNPs_with_called_genotypes_from_non-imputed = these are genotypes newly added by imputation).

| nAncestralHaplotypes | nGen | nDiff_from_non-imputed | nMatch_from_non-imputed | nMissing_from_non-imputed | nAdditional_SNPs_with_called_genotypes_from_non-imputed |
|---|---|---|---|---|---|
| 14 | 100000 | 36 | 1767 | 356 | 3057 |
| 8 | 1000 | 52 | 1765 | 342 | 3015 |
| 8 | 10000 | 37 | 1765 | 357 | 3010 |
| 8 | 100000 | 33 | 1757 | 369 | 2977 |
| 10 | 100000 | 35 | 1753 | 371 | 3013 |
| 10 | 10000 | 35 | 1752 | 372 | 3035 |
| 14 | 1000 | 41 | 1748 | 370 | 3077 |
| 10 | 1000 | 43 | 1741 | 375 | 3060 |
| 12 | 10000 | 39 | 1741 | 379 | 3052 |
| 14 | 10000 | 46 | 1739 | 374 | 3044 |
| 12 | 100000 | 34 | 1722 | 403 | 3041 |
| 6 | 100000 | 48 | 1721 | 390 | 2903 |
| 12 | 1000 | 48 | 1715 | 396 | 3092 |
| 14 | 100 | 46 | 1707 | 406 | 3197 |
| 6 | 1000 | 43 | 1698 | 418 | 2977 |
| 10 | 100 | 48 | 1693 | 418 | 3102 |
| 6 | 10000 | 42 | 1685 | 432 | 2952 |

| | | | | | |
|---|---|---|---|---|---|
| 6 | 100 | 65 | 1683 | 411 | 3031 |
| 8 | 100 | 53 | 1673 | 433 | 3079 |
| 12 | 100 | 53 | 1671 | 435 | 3122 |
| 4 | 1000 | 58 | 1659 | 442 | 2742 |
| 4 | 10000 | 69 | 1630 | 460 | 2772 |
| 4 | 100 | 103 | 1615 | 441 | 2776 |
| 4 | 100000 | 71 | 1581 | 507 | 2715 |

**Table S5 Statistical analysis of observed heterozygosity (*H*O) for immune response gene groups in genes, exons and introns.** Means and standard deviations are shown for genes, exon and introns separately. Results are only presented for gene, intron and exon *H*O as only these showed significance for both ANOVA and posthoc correction with Benjamini-Hochberg (BH). Gene groups with different letters ('a' and 'b') indicate groups that had significantly different means whilst the same letters indicate non-significant different means.

**Gene *H*O**

| Granzyme | Interleukin | Killer_Cell | MHC_I | MHC_II | TLR | Other_IR |
|---|---|---|---|---|---|---|
| ab | ab | a | b | ab | ab | ab |

| | **Group** | ***H*O mean** | **St. Deviation** |
|---|---|---|---|
| **1** | Granzyme | 0.292 | 0.070 |
| **2** | Interleukin | 0.265 | 0.097 |
| **3** | Killer_Cell | 0.338 | 0.075 |
| **4** | MHC_I | 0.161 | 0.106 |
| **5** | MHC_II | 0.258 | 0.131 |
| **6** | TLR | 0.256 | 0.118 |
| **7** | Other_IR | 0.266 | 0.088 |

**Exon *Ho***

| Granzyme | Interleukin | Killer_Cell | MHC_I | MHC_II | TLR | Other_IR |
|---|---|---|---|---|---|---|
| ab | ab | a | b | ab | ab | b |

| | **Group** | ***H*O mean** | **St. Deviation** |
|---|---|---|---|
| **1** | Granzyme | 0.056 | NA |
| **2** | Interleukin | 0.210 | 0.065 |
| **3** | Killer_Cell | 0.319 | 0.098 |
| **4** | MHC_I | 0.155 | 0.105 |
| **5** | MHC_II | 0.274 | 0.132 |
| **6** | TLR | 0.243 | 0.131 |
| **7** | Other_IR | 0.293 | 0.088 |

**Intron *H*O**

| Granzyme | Interleukin | Killer_Cell | MHC_I | MHC_II | TLR | Other_IR |
|---|---|---|---|---|---|---|
| ab | ab | a | b | ab | ab | ab |

| | **Group** | ***H*O mean** | **St. Deviation** |
|---|---|---|---|
| **1** | Granzyme | 0.296 | 0.066 |
| **2** | Interleukin | 0.285 | 0.099 |
| **3** | Killer_Cell | 0.327 | 0.070 |
| **4** | MHC_I | 0.173 | 0.098 |
| **5** | MHC_II | 0.275 | 0.117 |
| **6** | TLR | 0.269 | 0.095 |
| **7** | Other_IR | 0.260 | 0.092 |

**Table S6**. **Observed ($H_O$) and expected ($H_E$) heterozygosity in genes, exons and introns in MERS-CoV positive (n = 36) and negative (n = 65) individuals.** *P*-values of mean differences were calculated with Welch *t* test.

| | **Genes_$H_O$** | **Genes_$H_E$** | **Exons_$H_O$** | **Exons_$H_E$** | **Introns_$H_O$** | **Introns_$H_E$** |
|---|---|---|---|---|---|---|
| **Negative** | 0.26 | 0.28 | 0.25 | 0.27 | 0.26 | 0.29 |
| **Positive** | 0.27 | 0.28 | 0.27 | 0.27 | 0.28 | 0.28 |
| ***p*-value** | 0.50 | 0.79 | 0.58 | 0.96 | 0.42 | 0.78 |
| **Welch *t*** | -0.68 | 0.27 | -0.55 | 0.05 | -0.80 | 0.28 |
| **df** | 180.29 | 183.97 | 137.67 | 139.77 | 162.80 | 165.79 |

**Table S7**. **Linkage Disequilibrium-based haplotype (gene-set) test showing 20 genes with significant SNPs at *p* < 0.05.** Identified candidate genes *MAGOHB*, *HLA-A-24*-like, *HLA-DPB1*-like, *DNAH7* and *PTPN4* are highlighted in bold. *HLA-A-24*-like and *MAGOHB* were nominally significant ($p < 0.05$) indicated with an asterisk. NSNP - Number of SNPs in set; NSIG - Total number of SNPs below *p*-value threshold; ISIG - Number of significant SNPs also passing LD-criterion; STAT - Average test statistic based on ISIG SNPs; EMP1 - Empirical set-based *p*-value; SNPs - positions of SNPs in the set.

| SET | NSNP | NSIG | ISIG | EMP1 | SNPs | Name | Description |
|---|---|---|---|---|---|---|---|
| Cadr_00029272 | 12 | 5 | 1 | 0.008* | chr34:15362634 | ***MAGOHB*** | Protein mago nashi homolog 2 (Bos taurus OX=9913) |
| Cadr_00022140 | 112 | 14 | 2 | 0.031* | chr20:23100696 \| 23100503 | ***HLA-A-24-like*** | HLA class I histocompatibility antigen, A-24 alpha chain (Homo sapiens OX=9606) |
| Cadr_00001327 | 32 | 1 | 1 | 0.032* | chr2:5565157 | *Suclg1* | Succinate--CoA ligase [ADP/GDP-forming] subunit alpha, mitochondrial (Mus musculus OX=10090) |
| Cadr_00022016 | 41 | 9 | 1 | 0.058 | chr20:20681619 | ***HLA-DPB1-like*** | HLA class II histocompatability antigen, DP alpha chain (Homo sapiens OX=9606) |
| Cadr_00028914 | 14 | 7 | 2 | 0.060 | chr33:12210072 \| 12210460 | *IL10RA* | Interleukin-10 receptor subunit alpha (Homo sapiens OX=9606) |
| Cadr_00011189 | 2 | 1 | 1 | 0.063 | chr9:33578918 | *HP* | Haptoglobin (Sus scrofa OX=9823) |
| Cadr_00029993 | 12 | 2 | 2 | 0.085 | chr28:10593199 \| 10591217 | *NKL* | Antimicrobial peptide NK-lysin (Fragment) (Sus scrofa OX=9823) |
| Cadr_00001384 | 10 | 1 | 1 | 0.093 | chr2:9866197 | *RNF175* | RING finger protein 175 (Homo sapiens OX=9606) |
| Cadr_00001385 | 10 | 1 | 1 | 0.093 | chr2:9866197 | *TLR2* | Toll-like receptor 2 (Equus caballus OX=9796) |
| Cadr_00017035 | 3 | 1 | 1 | 0.095 | chr12:24456808 | *IFNG* | Interferon gamma (Camelus bactrianus OX=9837) |
| Cadr_00020479 | 129 | 21 | 4 | 0.117 | chr:1723840747 \| 23963829 \| 23948208 \| 23854332 | ***DNAH7*** | Dynein heavy chain 7 axonemal (Homo sapiens OX=9607) |
| Cadr_00006681 | 163 | 8 | 3 | 0.163 | chr5:8508361 \| 8569590 \| 8531515 | ***PTPN4*** | Tyrosine-protein phosphatase non-receptor type 4 (Homo sapiens OX=9606) |
| Cadr_00022139 | 67 | 6 | 2 | 0.188 | chr20:23039666 \| 23044464 | *Patr-A* | Patr class I histocompatibility antigen, A-126 alpha chain (Pan troglodytes OX=9598) |
| Cadr_00022038 | 34 | 1 | 1 | 0.220 | chr20:21059892 | *Mamu-DRA* | Mamu class II histocompatibility antigen, DR alpha chain (Macac mulata OX=9544) |
| Cadr_00022027 | 28 | 1 | 1 | 0.260 | chr20:20837533 | *BoLA-DQB* | BoLa class II histocompatability antigen, DQB*0101 beta chain (Bos taurus OX=9913) |
| Cadr_00029273 | 19 | 1 | 1 | 0.278 | chr34:15371765 | *Klra2* | Killer cell lectin-like receptor 2 (Mus musculus OX=10090) |

| | | | | | | | |
|---|---|---|---|---|---|---|---|
| Cadr_00022145 | 30 | 2 | 1 | 0.314 | chr20:23134732 | *HLA-A-11-like* | HLA class I histocompatibility antigen, A-11 alpha chain (Homo sapiens OX=9606) |
| Cadr_00022026 | 72 | 3 | 1 | 0.345 | chr20:20830433 | *HLA-DOB* | HLA class II histocompatability antigen, DO alpha chain (Homo sapiens OX=9606) |
| Cadr_00030053 | 90 | 2 | 2 | 0.427 | Contig45:329958 \| 334608 | *DDX58* | Probable ATP-dependent RNA helicase DDX58 (Sus scrofa OX=9823) |
| Cadr_00002239 | 297 | 17 | 3 | 0.427 | chr2:113136710 \| 113141381 \| 113168889 | *CC2D2A* | Coiled-coil and C2 domain-containing protein 2A (Homo sapiens OX=9606) |
